# A Graphene Composite Film Based Wearable Far‐Infrared Therapy Apparatus (GRAFT) for Effective Prevention of Postoperative Peritoneal Adhesion

**DOI:** 10.1002/advs.202309330

**Published:** 2024-03-25

**Authors:** Xiaohuan Lu, Luming Xu, Yu Song, Xiangnan Yu, Qilin Li, Feng Liu, Xiaoqiong Li, Jiangbo Xi, Shuai Wang, Lin Wang, Zheng Wang

**Affiliations:** ^1^ Hubei Key Laboratory of Regenerative Medicine and Multi‐disciplinary Translational Research Union Hospital Tongji Medical College Huazhong University of Science and Technology Wuhan 430022 China; ^2^ Hubei Provincial Engineering Research Center of Clinical Laboratory and Active Health Smart Equipment Union Hospital Tongji Medical College Huazhong University of Science and Technology Wuhan 430022 China; ^3^ Research Center for Tissue Engineering and Regenerative Medicine Union Hospital Tongji Medical College Huazhong University of Science and Technology Wuhan 430022 China; ^4^ Department of Gastrointestinal Surgery Union Hospital Tongji Medical College Huazhong University of Science and Technology Wuhan 430022 China; ^5^ Department of Clinical Laboratory Union Hospital Tongji Medical College Huazhong University of Science and Technology Wuhan 430022 China; ^6^ Department of Gastrointestinal Surgery The First Affiliated Hospital of Nanchang University Nanchang 330006 China; ^7^ School of Chemistry and Environmental Engineering Wuhan Institute of Technology Wuhan 430205 China; ^8^ Key Laboratory of Material Chemistry for Energy Conversion and Storage of Ministry of Education Department of Chemistry and Chemical Engineering Huazhong University of Science and Technology Wuhan 430074 China

**Keywords:** apparatus, far‐infrared, graphene, postoperative peritoneal adhesion

## Abstract

Postoperative peritoneal adhesion (PPA) is the most frequent complication after abdominal surgery. Current anti‐adhesion strategies largely rely on the use of physical separating barriers creating an interface blocking peritoneal adhesion, which cannot reduce inflammation and suffers from limited anti‐adhesion efficacy with unwanted side effects. Here, by exploiting the alternative activated macrophages to alleviate inflammation in adhesion development, a flexible graphene‐composite‐film (F‐GCF) generating far‐infrared (FIR) irradiation that effectively modulates the macrophage phenotype toward the anti‐inflammatory M2 type, resulting in reduced PPA formation, is designed. The anti‐adhesion effect of the FIR generated by F‐GCF is determined in the rat abdominal wall abrasion‐cecum defect models, which exhibit reduced incidence and area of PPA by 67.0% and 92.1% after FIR treatment without skin damage, significantly superior to the clinically used chitosan hydrogel. Notably, within peritoneal macrophages, FIR reduces inflammation reaction and promotes tissue plasminogen activator (t‐PA) level via the polarization of peritoneal macrophages through upregulating Nr4a2 expression. To facilitate clinical use, a wirelessly controlled, wearable, F‐GCF‐based FIR therapy apparatus (GRAFT) is further developed and its remarkable anti‐adhesion ability in the porcine PPA model is revealed. Collectively, the physical, biochemical, and in vivo preclinical data provide compelling evidence demonstrating the clinical‐translational value of FIR in PPA prevention.

## Graphical abstract

A graphene composite film‐based wearable and flexible far‐infrared therapy apparatus effectively prevents postoperative adhesion by regulating the Nr4a2 expression and promoting M2 macrophage polarization.

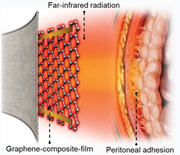

## 1 Introduction

Despite the significant progress in surgical techniques and devices, postoperative peritoneal adhesion (PPA) is still the most frequent complication after intra‐abdominal operations, reportedly occurring in up to 90% of patients.^[^ ^1^ ^]^ PPA leads to several adverse events, including intestinal obstruction, chronic pain, infertility, and even increased mortality risk due to repeated surgeries caused by adhesiolysis.^[^ ^2^ ^]^ To prevent PPA, one of the major options is to form an anti‐adhesion interface by applying specific reagents (such as liquid solutions, solid films, or hydrogels) into the abdominal cavity.^[^ ^2^, ^3^ ^]^ However, their anti‐adhesion effects are challenged by several limitations. The high absorption capacity of the peritoneum (1–2 mL min^−1^) results in liquid solutions being completely absorbed within 1–2 days, temporally insufficient to cover the critical period of adhesion development.^[^ ^2^, ^4^ ^]^ Solid films are inconvenient to be applied as they require meticulous hemostasis and effective suturing.^[^ ^2^, ^5^ ^]^ Although anti‐adhesive hydrogels have been widely used for preventing adhesion,^[^ ^6^ ^]^ the complete coverage of injured tissues and anti‐adhesion effects are far from ideal.^[^ ^7^ ^]^ Thus, new strategies are highly desired to efficiently prevent PPA.

The formation of PPA is a highly complex process involving peritoneal injury healing, inflammation responses, activation of the coagulation cascade, infection, and so on.^[^ ^2^, ^8^ ^]^ Pro‐inflammatory processes, such as macrophage activation, reportedly play an important role in adhesion formation.^[^ ^8^ ^]^ M1 macrophages (classical macrophages) can cause oxidative stress on the mesothelium, and secrete several proinflammatory cytokines, including interleukin‐1α (IL‐1α), interleukin‐1β (IL‐1β), and tumor necrosis factor‐α (TNF‐α), which promote the formation of adhesion.^[^ ^9^ ^]^ The activated Rho kinase pathway in M1 macrophages leads to an upregulation of plasminogen activator inhibitor (PAI‐1), which in turn inhibits plasminogen activation and promotes adhesion formation. By contrast, the infiltrated M2 macrophages (alternatively activated macrophages) play an anti‐inflammatory role at the late stages of inflammation, promoting the wound healing process of surgical injuries, and are associated with diminished adhesion formation.^[^ ^2c^ ^]^ It was reported that peroxisome proliferator‐activated receptor‐γ (PPAR‐γ) agonism reduced inflammation and induced a shift toward macrophage M2 polarization, thus preventing adhesion formation.^[^ ^10^ ^]^ Therefore, we hypothesized that it might be a promising strategy for preventing postoperative adhesion formation by regulating macrophage polarization.

Far‐infrared (FIR), an electromagnetic wave with a wavelength between 3 and 1000 µm, brings a variety of biological effects to the human body, especially in the wavelength range of 4–14 µm.^[^ ^11^ ^]^ FIR is currently being investigated as a potential therapeutic strategy for various diseases, including wound healing, cardiovascular diseases, chronic kidney diseases, and cancer.^[^ ^12^ ^]^ Several studies have revealed the anti‐inflammatory effect of FIR.^[^ ^13^ ^]^ For instance, FIR inhibited interleukin‐6 (IL‐6) and TNF‐α activity in mice with peritonitis.^[^ ^13a^ ^]^ In hemodialysis patients, FIR inhibited vascular endothelial inflammation via stimulating the expression of heme oxygenase 1,^[^ ^13b^ ^]^ and reduced inflammatory responses by suppressing proinflammatory M1 macrophages’ polarization via regulating the activity of NLRP3 (NOD‐, LRR‐ and pyrin domain‐containing 3) inflammasomes.^[^ ^12^, ^14^ ^]^ This anti‐inflammatory property of FIR may be utilized for the regulation of postoperative inflammatory responses toward preventing the formation of adhesion. Notably, FIR can penetrate ≈4 cm‐thick abdominal wall,^[^ ^11a^ ^]^ providing a premise for treating peritoneal diseases. Its therapeutic effect has been shown in treating encapsulating peritoneal sclerosis and improving the peritoneal membrane function of peritoneal dialysis patients.^[^ ^15^ ^]^ The aforementioned FIR's biological effects support the notion that FIR might prevent PPAs by suppressing post‐injury inflammatory responses.

However, the commonly used FIR sources, halogen lamps or ceramics,^[^ ^11a^ ^]^ require a high power input to generate FIR with a sufficiently high intensity. Their safety concerns, such as electric shock, skin burning, and heat shock resulting from high temperature, limit their clinical application.^[^ ^12e^ ^]^ In addition, these devices are usually unportable, thus bringing inconvenience to patients when long‐term therapy is required. Recently, graphene film‐based FIR generators have shown unique properties in biomedical applications. FIR generated by the graphene film was easily absorbed by the human body, as its radiation's emission peak position matched the characteristic absorption peaks of the human body, thereby avoiding skin burn induced by the absorbed infrared at other wavelengths.^[^ ^11^, ^12^ ^]^ Moreover, the high flexibility of graphene film makes it readily integrated into a wearable device, providing preferable convenience for clinical use.

**Scheme 1:**
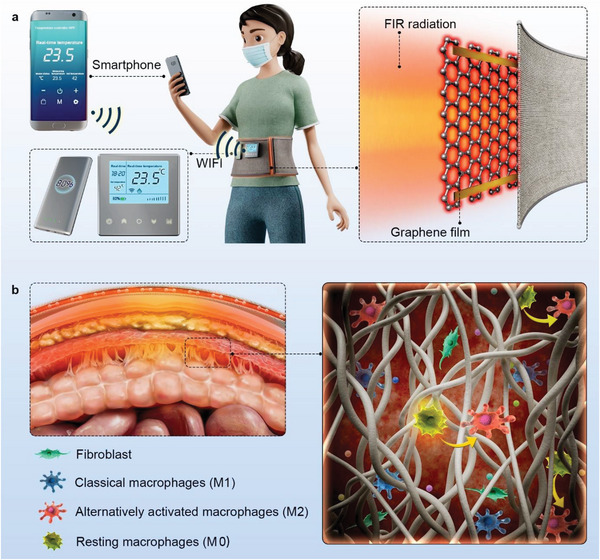
Schematic illustrations of a smartphone integrated with a flexible graphene‐composite‐film (F‐GCF) device for preventing postoperative peritoneal adhesion. a) Schematic illustration of a working wirelessly controlled wearable FIR therapy apparatus and a smartphone. b) FIR effectively prevented the formation of PPA via modulating macrophages’ polarization.

In this study, we fabricated a flexible graphene‐composite film (F‐GCF) that could generate FIR under a low‐voltage power supply and evaluated the performance of FIR in preventing PPA (**Scheme** **1**). We found that FIR effectively prevented the formation of PPA via modulating macrophage polarization. Furthermore, based on F‐GCF, we designed and presented a wirelessly controlled, wearable FIR therapy apparatus (GRAFT), and confirmed its anti‐adhesion efficacy in a porcine preclinical model. Together, our results demonstrate that the graphene‐generated FIR treatment is a promising strategy for preventing PPA.

## 2 Results and Discussion

### 2.1 Fabrication and Characterizations of the FIR Generatable Graphene Film

**Figure 1:**
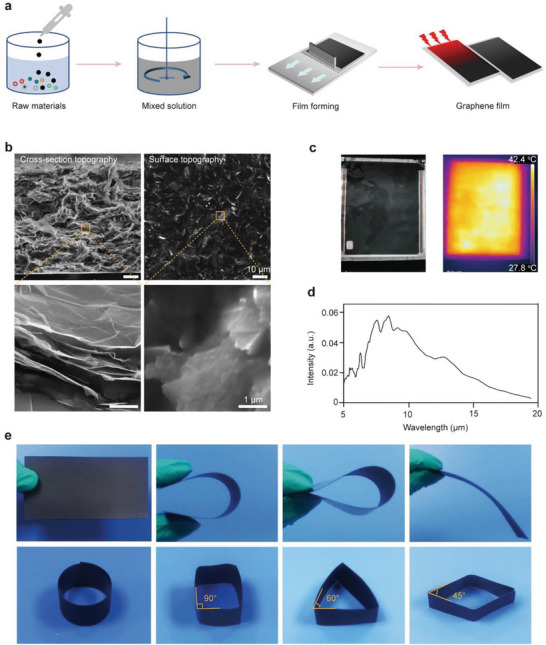
Fabrication and characterization of the FIR‐generatable graphene composite film. a) Schematic diagram of the fabrication process of F‐GCF. b) SEM images of the cross‐section and surface topography of graphene film. Scale bars, 10 and 1 µm. c) The photograph and thermal imaging graph of a 30 cm × 35 cm F‐GCF with a 45 V power input. d) Far‐infrared emission spectra of F‐GCF. e) Photographs showing the flexible property of F‐GCF.

The fabrication process of the FIR generatable graphene‐composite‐film (F‐GCF) was schematically shown in **Figure** **1**a and included three steps: 1) mixing the graphene with the conductive graphite, strength intensifiers, and adhesives; 2) spreading the mixture on a polyethylene terephthalate membrane and rolling it into a 0.1 cm thick film using a three‐high mill; 3) installing copper electrodes on both ends of the film after drying at 120 °C. The scanning electron microscopy (SEM) images showed that the graphene sheets were uniformly distributed in F‐GCF (Figure 1b). The electric‐thermal radiation conversion behavior of F‐GCF exhibited a power intensity‐dependent manner (Figure S1a, Supporting Information); and its efficiency was measured and calculated to be 83%. After applying a voltage (45 V), the temperature of F‐GCF was increased by 14.6 °C within 5 min (Figure 1c; Figure S1b, Supporting Information). Continuous monitoring of temperature demonstrated that F‐GCF can maintain stable working at a temperature of 42 °C for a duration of at least 20 days (Figure S2, Supporting Information). The emission wavelength of F‐GCF ranged from 5–19.5 µm with a peak at 8.5 µm (Figure 1d), close to the FIR wavelength range of human tissues (8–14 µm),^[^ ^11a^ ^]^ indicating that the FIR generated by F‐GCF could penetrate deeply into the tissue. We measured the tissue penetration capacity of F‐GCF generating FIR and found that the heat effect of the FIR could reach a depth of nearly 5 cm (Figure S1c,d, Supporting Information). Moreover, F‐GCF had a satisfactory flexible property, with the ability to bend up 45° without breaking (Figure 1e; Video S1, Supporting Information), suggesting its potential suitability for use in wearable devices.

### 2.2 FIR Treatment Reduces Postoperative Peritoneal Adhesion Formation In Vivo

**Figure 2:**
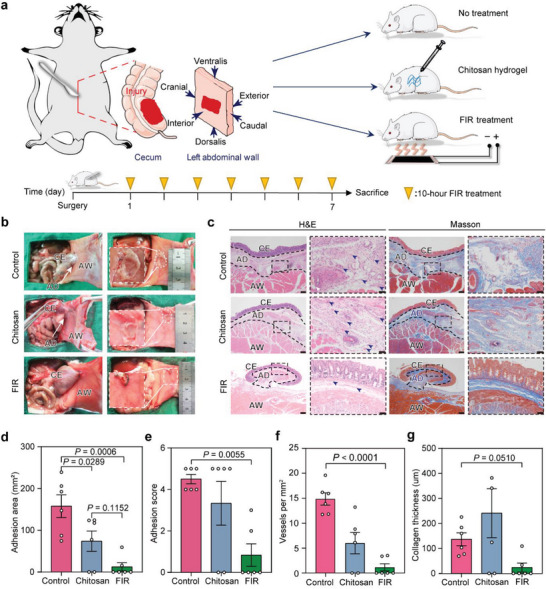
FIR prevents postoperative peritoneal adhesion formation in the rat model. a) The experimental flowchart of investigating the in vivo adhesion‐preventing efficacy of FIR. Rats without treatment or treated with chitosan hydrogel injection were set as controls. The timeline shows that the rats were treated by FIR for 10 h per day on post‐operation days 1–7. b) Representative photographs of the postoperative peritoneal adhesion (PPA) in each group on post‐operation day 7. The white dashed lines indicate the boundaries of adhesion. CE: cecum; AW: abdominal wall; AD: adhesion. c) Representative hematoxylin and eosin (H&E) and Masson staining images of the adhesion tissues in different groups on post‐operation day 7. The black dashed lines indicate the boundaries of adhesion; the blue arrows indicate blood vessels. Scale bars, 200 µm for the original images, and 50 µm for the enlarged images. d) The qualification of adhesion areas of each group on post‐operation day 7. e) Adhesion scores of different groups on post‐operation day 7. f) The vessel number in the adhesion areas on post‐operation day 7. g) The quantification of collagen deposition thicknesses. Data are shown as mean ± SD; six rats per group; *p* values were calculated using a one‐way ANOVA test.

The capability of the FIR generated by F‐GCF in preventing PPA formation was first investigated in a cecum abrasion‐abdominal wall defect rat model (Figure S3, Supporting Information),^[^ ^3c,d^ ^]^ in which the injured cecum was sutured to the defected peritoneal wall to force adhesion formation during laparotomy. The rats were treated by FIR irradiation for seven days (10 h per day), with an F‐GCF temperature of 42 °C (**Figure** **2**a). Histologically, such a treatment intensity could not induce chronic burns on the abdomens of rats (Figure S4, Supporting Information). The rats without any treatment or injected with the commercially used chitosan hydrogel (CHIOGEL, chitosan group) were set as negative controls and positive controls, respectively (Figure 2a). We measured and scored (see **Table** **1** in Experimental Section) the adhesion between the abdominal wall and cecum on day 7 after the operation. Compared to no treatment and chitosan hydrogel, FIR treatment reduced the incidence of PPA by 66.7% and 50.0%, respectively (Figure 2b–d). All the untreated rats formed severe adhesion (grade‐4 or −5) that could not be bluntly separated (Figure 2b–e). The adhesion area of untreated rats reached 157.7 mm^2^ (Figure 2d), with obvious vascularization and collagen deposition (Figure 2f,g). Although chitosan hydrogel injection reduced the average adhesion area to 73.8 mm^2^, the highest level (grade‐5) adhesion was still formed in four out of six rats (66.7%, Figure 2c–e; Figure S5, Supporting Information). By contrast, FIR treatment remarkably prevented the formation of adhesion: only two of six FIR‐treated rats (33.0%) formed thin and easily separable adhesions (grade‐2 or −3 adhesion, respectively) with an average area of 12.5 mm^2^ (Figure 2d,e; Figure S5, Supporting Information), significantly lower than the untreated rats and chitosan‐treated rats. Further, the deposition of collagen and angiogenesis were also reduced in the FIR‐treated rats (Figure 2f,g).

**Table 1:** Extent of adhesions scoring scale.

Grade	Rat model	Porcine model
5	More than one plantar adhesion or very thick vascularized adhesion	/
4	More than one thick adhesion with a focal point or thick adhesion with a plantar attachment	Fibrotic, plain fused adhesions forming a bowel package, separation not possible without injury of the organ (transintestinal dissection)
3	Thick adhesions with a focal point	Broad strains and plain adhesions, considerable sharp dissection
2	More than one thin filmy adhesion	Beginning vascularization, filiform, or broad strains, blunt and some sharp dissection
1	One thin filmy adhesion	Apposition of fibrin, filiform strains, and loose, filmy adhesions and blunt dissection
0	No adhesion	Clean, no adhesions

We next investigated whether the adhesion‐preventing ability of FIR irradiation was only caused by the thermal effect. An opaque aluminum foil (≈10 µm thickness) was covered on the surface of F‐GCF (Figure S6a, Supporting Information), acting as an FIR shield, which can block the FIR irradiation generated by F‐GCF, and did not affect the temperature rise caused by heat conduction (Figure S1b, Supporting Information).^[^ ^12^, ^16^ ^]^ Compared with the untreated rats, the adhesion area of the rats treated by heat alone was slightly decreased (Figures S5b,e and S7, Supporting Information), which might be caused by the heat‐induced intestinal peristalsis enhancement.^[^ ^17^ ^]^ However, all the heat‐treated rats also formed grade‐4 or −5 adhesions (Figure S6c, Supporting Information). Meanwhile, similar to untreated rats, the formation of tight adhesions and thick collagen depositions between the worn cecum and damaged abdominal wall was also observed in all heat‐treated rats (Figure S6d,f, Supporting Information). These results suggest that heating alone cannot effectively prevent PPA formation.

The long‐term (10 weeks) adhesion‐preventing effect of FIR irradiation was further evaluated. After establishing the cecum abrasion‐abdominal wall defect rat model, the rats were treated with FIR for 7 days (10‐h FIR radiation per day, with an F‐GCF temperature of 42 °C), then sacrificed 70 days after the operation (Figure S8a, Supporting Information). The anatomical and histological examinations showed that the abdominal wall and cecum of all the rats in the control group were completely bonded together by adhesion tissues on day 70 (Figures S8b–d and S9, Supporting Information). The adhesion's average area and thickness, respectively, reached 120.5 mm^2^ and 336.0 µm (Figure S8e,f, Supporting Information), with the corresponding grades up to four or five (Figure S8g, Supporting Information). In contrast, the peritoneum of the FIR‐treated group was healed with a clearly visible mesothelial monolayer (Figure S8b,c, Supporting Information); and only two rats left a few adhesive strips (Figures S8h and S10, Supporting Information). The body weights of the FIR‐treated rats grew significantly higher than that of the untreated rats (Figure S8i, Supporting Information), suggesting that the reduced adhesions improve gastrointestinal functions. Overall, these results indicate that FIR irradiation prevents PPA formation in the cecum abrasion‐abdominal wall defect rat model.

### 2.3 FIR Reduces Adhesion Formation Possibly Through Modulating Inflammatory Responses

Next, we delved into the mechanism through which FIR inhibited the formation of PPA. Since collagen deposition and vascular ingrowth are the main consequences of excessive fibrinous exudation that were elicited by the local inflammatory responses after the injury of the peritoneum and bowel wall,^[^ ^18^ ^]^ we assessed whether FIR repressed adhesion formation through regulating inflammatory responses. Inflammatory cells (macrophages stained by CD68; neutrophils stained by myeloperoxidase) in the adhesion tissue (or the injured site of the abdominal wall, for rats without adhesion formation) were analyzed during treatments (Figure S11a,b, Supporting Information). On post‐operation days 1, 3, and 7, the numbers of infiltrated macrophages and neutrophils in the adhesions of FIR‐treated rats were ≈5.3–8‐fold (macrophages) and 7–10‐fold (neutrophils) less than those in untreated rats, respectively (Figures S11e,f and S12a–d, Supporting Information). The fewer number of TNF‐α or vascular endothelial‐derived growth factor (VEGF, key factors for inflammatory response and angiogenesis, respectively) positive cells were also observed on post‐operation days 1, 3, and 7 (Figures S11c,d and S12e,h, Supporting Information). These results suggest that FIR might modulate inflammation responses during adhesion formation.

**Figure 3:**
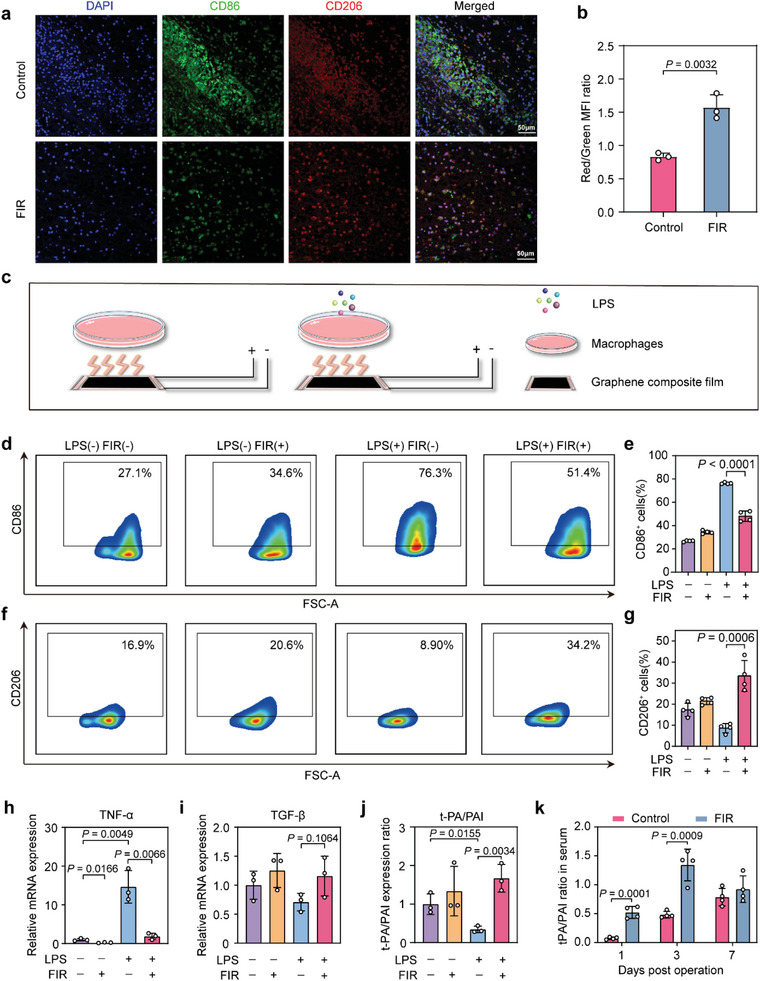
FIR modulates the polarization of peritoneal macrophage in vivo and in vitro. a) Representative immunofluorescence staining images show the M1 and M2 macrophages in the adhesion tissues. CD86 (green) or CD206 (red) positive cells were determined as M1 or M2 macrophages, respectively. b) M2 / M1 macrophage ratio was determined by red‐to‐green fluorescence intensity ratio in the immunofluorescence staining results. c) Schematic diagram of the in vitro experiment design. The rat primary peritoneal macrophages were cultured in the medium with or without 5 µg mL^−1^ lipopolysaccharide (LPS, acting to induce the activation of macrophages) for 6 h, then treated by FIR irradiation (37 °C) for 30 min. d,e) The percentage of CD86 positive macrophages in rat primary peritoneal macrophages with the indicated treatments was determined using flow cytometry. f,g) The percentage of CD206 positive macrophages in rat primary peritoneal macrophages with the indicated treatments was determined using flow cytometry. h,i) Relative mRNA expression level of h) TNF‐α and i) TGF‐β in rat primary peritoneal macrophages with the indicated treatments. j) The mRNA expression ratio of t‐PA and PAI‐1 in rat primary peritoneal macrophages with the indicated treatments. k) The protein concentration ratio of serum t‐PA and PAI‐1 in the untreated or FIR‐treated cecum abrasion‐abdominal wall defect rats on post‐operation days 1, 3, and 7. The serum concentrations of t‐PA and PAI‐1 were measured by ELISA. Data are shown as mean ± SD; *n* = 4 for the flow‐cytometry and ELISA experiments; *n* = 3 for other experiments; *p* values were calculated using a two‐tailed Student's *t*‐test.

Macrophage polarization plays a crucial role in regulating inflammatory response. We then determined whether FIR affected macrophages’ polarization during adhesion formation. Macrophages can convert into pro‐inflammatory M1 phenotype (CD86^+^) or anti‐inflammatory M2 phenotype (CD206^+^) in response to specific pathophysiological stress. Within the FIR‐treated site, the number of CD206^+^ cells was significantly higher than that of CD86^+^ cells (**Figure** **3**a,b), suggesting that FIR drives macrophage polarization toward the M2 phenotype. To further verify this FIR‐mediated macrophage polarization, primary rat peritoneal macrophages (resting macrophages) were initially activated with lipopolysaccharide (LPS) that often drives macrophage polarization toward pro‐inflammatory M1 phenotype (Figure 3c). Notably, the subsequent FIR irradiation significantly reduced the LPS‐treated macrophages’ M1‐like polarization (Figure 3d,e). The mean percentage of M1 macrophages (CD86+) in the LPS (+) FIR (+) group (48.1%) was significantly lower than that in the LPS (+) FIR (−) group (76.3%) (Figure 3d,e), accompanied by the increased M2 macrophage (CD206+) percentage (Figure 3f,g). A decrease of pro‐inflammatory cytokine TNF‐α and an increasing expression of healing‐promoting growth factor transforming growth factor‐β (TGF‐β) were also observed in the LPS‐treated, FIR‐irradiated macrophages (Figure 3h,i). Further, FIR treatment up‐regulated the relative mRNA expression ratio of tissue plasminogen activator (t‐PA) versus PAI‐1 (t‐PA/PAI‐1 ratio, reflects the level of fibrinolytic activity) in LPS‐treated primary peritoneal macrophages (Figure 3j). This observation was consistent with the increased serum protein concentration ratio of t‐PA/PAI‐1 in FIR‐treated cecum abrasion‐abdominal wall defect rats (Figure 3k), indicating that FIR treatment relieves the pro‐inflammatory macrophage‐mediated reduction of fibrinolytic activity. Overall, these results reveal that the effectiveness of FIR in preventing PPA is associated with the regulation of inflammatory responses.

### 2.4 FIR Modulates Peritoneal Macrophage Polarization Through Inducing Nr4a2 Expression

**Figure 4:**
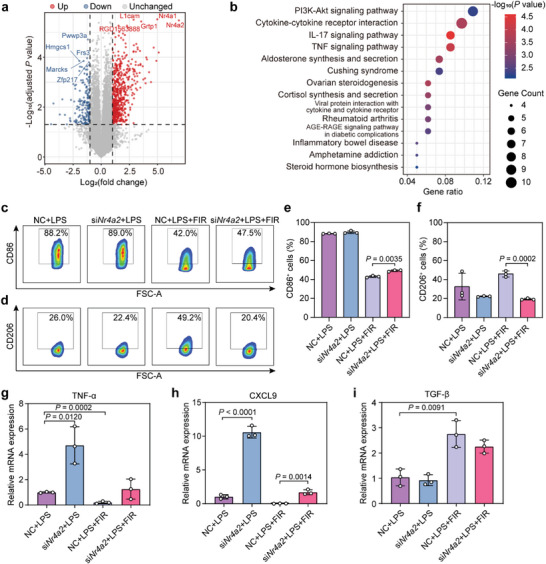
FIR promotes peritoneal macrophages Nr4a2 expression and induces M2 polarization. a) Volcano plot shows the differentially expressed genes (DEGs, |log_2_(fold change)| > 1 and adjusted *p* value < 0.05) in the LPS‐treated rat primary peritoneal macrophages with or without FIR irradiation. The symbols of the top 5 up‐regulated or down‐regulated genes were labeled. Four samples per group. b) KEGG pathway enrichment analysis of the DEGs in the LPS‐treated rat primary peritoneal macrophages with or without FIR irradiation. c,d) Representative flow cytometry results of the c) CD86 or d) CD206 positive macrophages in rat primary peritoneal macrophages with the indicated treatments. e,f) Quantification of the percentages of e) CD86 or f) CD206 positive macrophages from the flow cytometry results. g–i) Relative mRNA expression level of g) TNF‐α, h) CXCL9, and i) TGF‐β in rat primary peritoneal macrophages with the indicated treatments. Data are shown as mean ± SD; each flow cytometry or RT‐qPCR experiment was repeated three times; *p* values were calculated using a one‐way ANOVA test.

To further explore the influence of FIR treatment on macrophage polarization, primary peritoneal macrophages were subjected to RNA‐Seq and differential gene expression analysis. FIR significantly changed the gene expression pattern of LPS‐treated macrophages (**Figure** **4**a). KEGG pathway enrichment analysis showed that the differentially expressed genes (log_2_(fold change) > 1 and adjusted *p* value < 0.05) enriched in several immune‐related pathways, including PI3K‐Akt, interleukin‐17 (IL‐17) and TNF pathways (Figure 4b). Notably, the mRNA expression of two nuclear receptor 4A (Nr4a) family members, Nr4a1 and Nr4a2, was increased by 31.6‐ and 119‐fold in FIR‐treated macrophages, respectively (Figure 4a). Although both Nr4a1 and Nr4a2 reportedly suppress macrophage's pro‐inflammatory polarization,^[^ ^19^ ^]^ only Nr4a2 was found to have a significant increase at its protein level after FIR treatment (Figure S13, Supporting Information). Importantly, knockdown of Nr4a2 expression with its *siRNA* (si*Nr4a2*; Figure S14, Supporting Information) caused a drastic reduction in the percentage of M2 macrophages (CD206^+^) but an increase in M1 macrophages (CD86^+^; Figure 4c,e) in LPS‐treated, FIR‐treated primary peritoneal macrophages (Figure 4d,f), suggesting that the effect of FIR on macrophage polarization possibly depends on Nr4a2 expression. Consistently, knockdown of Nr4a2 also increased the expression of pro‐inflammatory cytokine TNF‐α and CXCL9 and reduced TGF‐β expression in LPS‐treated, FIR‐irradiated primary peritoneal macrophages (Figure 4g–i). These findings collectively suggest that FIR treatment reduces inflammation possibly by inducing Nr4a2 expression.

### 2.5 Up‐Regulated Nr4a2 Mediates FIR's Prevention on PPA

**Figure 5:**
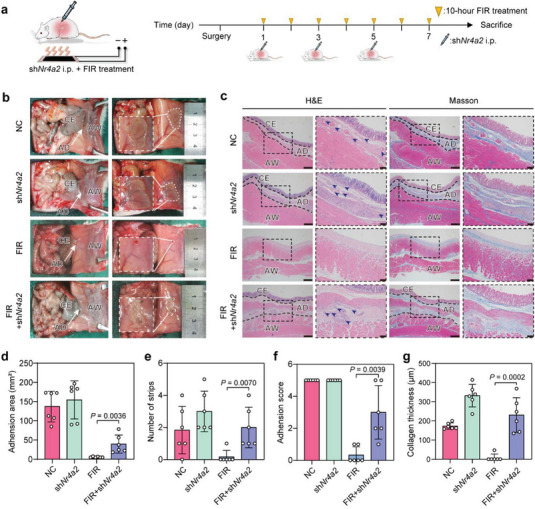
The postoperative peritoneal adhesion‐preventing ability of FIR is dependent on the up‐regulation of Nr4a2. a) Flowchart of the animal experiments. During the FIR‐treatment process, sh*Nr4a2* lentivirus was intraperitoneally injected into the rats every second day. b) Representative photographs of the PPA in each group on post‐operation day 7. The white dashed lines indicate the boundaries of adhesion. CE: cecum; AW: abdominal wall; AD: adhesion. c) Representative H&E and Masson staining images of the adhesion tissues in different groups on post‐operation day 7. The black dashed lines indicate the boundaries of adhesion; blue arrows indicate blood vessels. Scale bars, 200 µm for the original images, and 50 µm for the enlarged images. d,e) Qualification of d) adhesion areas, and e) adhesion score of each group on post‐operation day 7. f) The number of adhesion strips on post‐operation day 7. g) Quantification of collagen deposition thicknesses. Data are shown as mean ± SD; six rats per group; *p* values were calculated using a two‐tailed Student's *t*‐test.

The role of FIR‐induced up‐regulation of Nr4a2 in PPA formation was further investigated in vivo. We constructed a macrophage‐targeting lentivirus vector (pAV‐F4/80‐GFP‐miR30‐sh*Nr4a2*, sh*Nr4a2* lentivirus) to specifically inhibit the FIR‐induced Nr4a2 up‐regulation of peritoneal macrophage in rat models (**Figure** **5**a). After modeling, the rats were intraperitoneally injected (i.p.) with the sh*Nr4a2* lentivirus or a negative control (NC) lentivirus every other day and treated by FIR irradiation for seven consecutive days (10 h per day). Immunofluorescent staining showed that sh*Nr4a2* lentivirus significantly inhibited the FIR‐induced increase of Nr4a2 expression (Figure S15, Supporting Information). After a 7‐day FIR treatment, the average adhesion area and number of strips of rats with sh*Nr4a2* lentivirus injection, respectively, reached 39.3 mm^2^ and 2.0‐, 13‐ and 12‐fold more than the rats with NC lentivirus (3.0 mm^2^ and 0.17, respectively; Figure 5b–d; Figure S16, Supporting Information). Moreover, five out of six rats (5/6) with sh*Nr4a2* lentivirus injection developed grade‐2 or higher PPA, more serious than the rats with NC lentivirus (2/6; only two out of six rats developed grade‐1 PPA) (Figure 5e,f), suggesting that the suppression of Nr4a2 expression markedly reduces FIR's PPA‐preventing ability. In addition, H&E and Masson staining showed the formation of dense adhesions with neovascularization and substantial collagen deposition in sh*Nr4a2* lentivirus‐treated rats subsequently receiving FIR (Figure 5c,g). Notably, the inhibition of Nr4a2 expression could not completely reverse the preventive effect of FIR on PPA, suggesting that additional pathways might also play a role. These results collectively imply that the up‐regulation of Nr4a2 might be an important factor responsible for FIR's ability to modulate macrophage polarization and prevent PPA.

### 2.6 The Design and Fabrication of a Wirelessly Controlled Wearable FIR Therapy Apparatus

**Figure 6:**
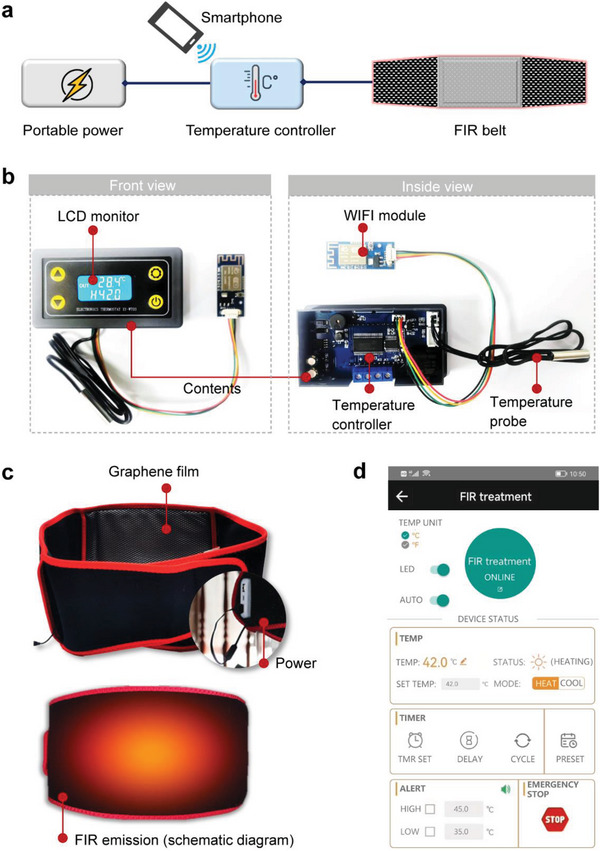
Overview of the wirelessly controlled and wearable FIR therapy apparatus (GRAFT). a) Schematic diagram of the FIR emission belt showing that the apparatus was composed of portable power, a wireless temperature controller, smartphone software for real‐time controlling and monitoring, and a wearable belt containing F‐GCF. b) The vertical view of the wirelessly controllable temperature controller. The control instructions and the real‐time temperature (obtained from the temperature probe) are transmitted with a smartphone through the WIFI module. c) The wearable belt that emits FIR. d) The screenshot of the control APP software's interface showing the main functions of the software, which enables the monitoring of treatment processes in real‐time, providing a visual display of treatment status.

Aiming at facilitating the convenient and precise control of FIR for effective anti‐adhesion, we designed and fabricated a wirelessly controlled, graphene‐composite‐film‐based FIR therapy apparatus (GRAFT, **Figure** **6**a). GRAFT included three components: 1) a wireless temperature controller that could adjust and monitor the film's temperature (Figure 6b); 2) a 120 cm × 17 cm wearable belt that contained a 20 cm × 15 cm F‐GCF (Figure 6c); 3) a smartphone software allowing wireless control of the temperature controller (Figure 6d). The temperature controller transmitted the real‐time temperature information (captured by a probe, also displayed on the liquid crystal display (LCD) monitor), and received the instructions regarding treatment duration and intensity via wireless signals sent from or sent to the software (Figure S17 and Video S2, Supporting Information). Compared with the commonly used FIR radiators (such as halogen lamps and ceramic infrared radiators),^[^ ^20^ ^]^ GRAFT possesses several technical advantages. First, the low power requirements (10 W) and working temperature (42 °C) make GRAFT safer than halogen lamps and ceramic infrared radiators, which often need over 200 W of input power with a working temperature over 100 °C. Second, GRAFT is flexible and wearable, allowing patients movement during treatment, thus improving patient compliance. Third, clinicians can monitor and control GRAFT's parameters with the smartphone in a real‐time fashion, thus facilitating personalized treatment and minimizing potential risks. In this way, GRAFT enabled remote monitoring and control of the anti‐adhesion treatment process. And it might not only for preventing PPA, but also for the treatment of other diseases utilizing FIR therapy.

### 2.7 GRAFT Prevents the Postoperative Adhesion Formation in a Porcine PPA Model

**Figure 7:**
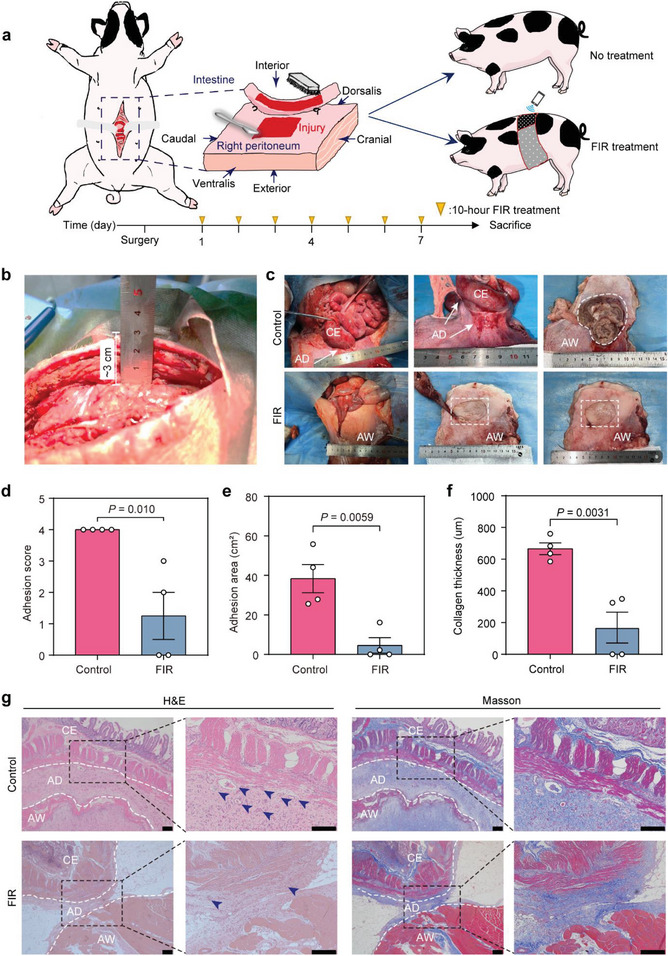
The adhesion‐preventing activity of GRAFT in the porcine PPA model. a) Schematic diagram of the experimental design and timeline. b) Representative photograph shows that the abdominal wall thickness is ≈3 cm. c) Representative photographs of the abdominal adhesions in FIR‐treated or untreated pigs on post‐operation day 7. d–f) The qualification of d) adhesion score, e) adhesion area, and f) collagen deposition thickness on post‐operation day 7. g) Representative H&E and Masson staining images of the adhesion tissues on post‐operation day 7. The white dotted lines indicate the boundaries of adhesion; blue arrows indicate blood vessels. CE: cecum; AW: abdominal wall; AD: adhesion. Scale bars, 200 µm for the original images, and 100 µm for the enlarged images. Data are shown as mean ± SD; four Bama miniature pigs per group; *p* values were calculated using a two‐tailed Student's *t*‐test.

The adhesion‐preventing effectiveness of GRAFT was investigated in a Bama miniature pig PPA model (**Figure** **7**a; Figure S18 and S19, Supporting Information). The abdominal wall thickness of a pig was ≈3 cm (Figure 7b), similar to that of an adult human. During the treatment process (10 h per day for 7 days), we placed GRAFT around the pig's abdomen and positioned F‐GCF directly against the peritoneal injury area (Figure S18 and Video S3, Supporting Information). The treatment temperature of GRAFT was remotely set to 42 °C using an Android smartphone, with an FIR emission intensity of 15 mW cm^−2^ (Video S4, Supporting Information). On day 7 after the establishment of the porcine surgical adhesion model (Figure S19, Supporting Information), the PPA incidence of GRAFT‐treated pigs was 50%, only half that of the untreated pigs (100%) (Figure 7c,d). All untreated pigs developed grade‐4 adhesions (highest level, see Table 1 in Experimental Section) between the small intestine and abdominal wall (Figure 7c,d; Figure S20, Supporting Information). Consistent with the findings in the rat model, the small intestines of all untreated pigs were tightly adhered to the abdominal walls, with an average adhesion area of 38.4 cm^2^ (Figure 7e) and a collagen deposition of 665.8 µm (Figure 7f,g). By contrast, although the injuries on the abdominal wall were not healed, the incidence and severity of adhesion significantly decreased after FIR treatment, with only two out of four pigs forming grade‐2 or grade‐3 adhesions. Compared with the untreated pigs, FIR dramatically reduced the adhesion area and collagen deposition thickness by 88% and 74.6%, respectively (Figure 7d,e). Together, these results revealed the effectiveness of GRAFT in PPA prevention.

## 3 Conclusion

In summary, we revealed the prevention efficacy of FIR on PPA by employing a flexible graphene composite film (F‐GCF) capable of generating FIR with a low‐voltage power supply. Moreover, we mechanically investigated the biological role of FIR in PPA prevention, found that FIR might drive macrophage polarization toward M2 phenotype through upregulating Nr4a2 expression, and provided a theoretical basis for FIR in treating more inflammatory diseases. Based on the F‐GCF, we presented a wirelessly controlled FIR therapy apparatus (GRAFT) and assessed its anti‐PPA efficacy in a porcine model. To the best of our knowledge, this is the first study that employed FIR to prevent PPA. In contrast to in vivo planting or injection of anti‐adhesive barriers, GRAFT is readily tunable and allows coverage of the entire operation area, thereby avoiding the treatment failures resulting from the displacement of anti‐adhesion materials. Moreover, the in vitro application of low‐strength FIR has long been considered a safe treatment approach, eliminating potential biosafety concerns linked to anti‐adhesive materials.

Collectively, our work demonstrated the potentiality of FIR for the prevention of PPA formation. Our findings provided a promising option for the prevention of postoperative complications based on in vitro intervention and offered a new wearable device for FIR's clinical applications.

## 4 Experimental Section

### Preparation of the F‐GCF

Few‐layered graphene nanosheet suspension (20.0 g, solid content 17%), conductive carbon black (20.0 g), poly (acrylate ammonium) (2.0 g), sodium dodecyl benzene sulfonate (2.0 g), and polysiloxane oil (2.0 g) were suspended in 200 mL DI water and subsequently mixed in a high‐speed homogenizer for 0.5 h with a speed of 15 000 rpm. The mixture was filtered to remove 90% water and denoted as a graphene composite suspension. Then, the graphene composite suspension was mixed with N‐containing flame retardant epoxy resin (50.0 g), P‐containing flame retardant epoxy resin (50. 0 g), curing agent (20.0 g), leveling agent (1.5 g), dispersant (1.0 g), and silicone defoaming agent (1.0 g) for 0.5 h (1000 rpm). The pH of the resultant suspension was adjusted to 8.5 with diethylenetriamine. The resultant mixture was denoted as composite resin suspension. The resultant graphene composite suspension (100.0 g), composite resin suspension (100.0 g), and silane coupling agent (1.0 g) were mixed in a high‐speed homogenizer for 1 h with a speed of 12 000 rpm. After being ground by a three‐roller grinder, a uniform, and fine far‐infrared composite graphene slurry was obtained. The slurry was then coated with the polyester film and baked in the oven at 120 °C for 24 h. The cupreous electrode layer was then pasted to obtain the F‐GCF. The thickness of F‐GCF can be adjusted in the range of 50–300 µm by changing the feeding amount of far‐infrared composite graphene slurry.

### Characteristics

The morphology of the F‐GCF was observed by scanning electron microscopy (SU8010, Hitachi, Tokyo, Japan). The emission wavelength was measured using a Fourier transform infrared spectrometer (Model No. Nicolet 6700, Thermo Electron Corp., Madison, WI). The thermograph was obtained by the FLIR One Pro system (Teledyne FLIR Co., Ltd. USA). According to Planck's and Stefan–Boltzmann radiation laws, the power emissivity (*ε*), and electric‐to‐radiant power transfer efficiency (*η*) of F‐GCF were detected and calculated by China National Infrared & Industrial Electrothermal Products Quality Supervision & Test Center (Wuhan, China).^[^ ^14b^ ^]^ The power emissivity (*ε*) of the sample was 0.89. The electric‐to‐radiant power transfer efficiency (*η*) of the sample was 83%.

The thermal effect and tissue penetration of the FIR radiation were tested in vitro. A digital thermometer (Model No. VC6801, Victor Instrument Co., Ltd., Xian, China) was used to measure the surface temperature of the geometrical center of F‐GCF (30 cm × 35 cm) that was increased by enlarging the output voltage. Finally, 45 V was selected for the subsequent experiments as the F‐GCF's surface temperature rapidly reached 42 °C under this condition which would not be harmful to human skin.^[^ ^12e^ ^]^ A pig abdominal wall with ≈5 cm thickness was put on the geometrical center of F‐GCF at room temperature, followed by the measurement of the temperature inside the tissue (0.5–5 cm of depth). The logical correlation between temperature (*T*) and tissue thickness (*H*) was *T* = −2.235*H* + 38.89 °C.

### Animals

SD rats (Male, 250 ± 20 g; Beijing SPF Laboratory Animal Biotechnology Co., Ltd. Beijing, China) and pigs (25 ± 3 kg; Hubei Yizhicheng Biotechnology Co., Ltd. Yingcheng, China) were raised in the specific pathogen‐free conditions at the Laboratory Animal Center of Tongji Medical College, Huazhong University of Science and Technology. This study was approved by the ethics committee of Tongji Medical College, Huazhong University of Science and Technology (Approval No. 2021‐S2287). All the animal experiments and protocols were approved by the Institutional Animal Care Committee of Huazhong University of Science and Technology, Wuhan, China.

### Rat Model of Surgical Adhesion Formation

Rats were anesthetized with pentobarbital sodium solution (20 mg mL^−1^). A midline incision (4 cm long) was made on the abdominal wall to expose the cecum, and the cecal serosa was abraded using a sterile gauze until the serosal surface was hemorrhagic. Next, a 2 cm × 1 cm peritoneum on the abdominal wall was removed using a surgical scissor; the injured cecum was sutured to the damaged abdominal wall with 4‐0 surgical silk. Finally, an abdominal incision was anastomosed with 4‐0 surgical silk sutures. For the chitosan hydrogel treated group, 0.6 mL of chitosan hydrogel (CHIOGEL, Shanghai Qisheng Biological Preparation Co., LTD) was injected into the injured abdominal wall and cecum before incision anastomosis. For the FIR‐treated group, the abdomens of the rats were exposed to FIR radiation for 7 days (10 h per day). For the thermally treated group, an aluminum foil was covered on the far‐infrared graphene composited film to block the FIR.

### Establishment of Porcine Postoperative Peritoneal Adhesion Model

Eight female pigs were raised in cages under standard laboratory conditions with a light/dark cycle of 12 h and randomly given food and water. The pigs were fasted for 12 h before surgery. The porcine model of surgical adhesion formation was established according to the previously reported method.^[^ ^21^ ^]^ In brief, the pigs were anesthetized by an initial intramuscular injection of 10 mg kg^−1^ ketamine (i.m.) and 0.05 mg kg^−1^ atropine and subsequently received intubation, continuous inhalation of 2%−3% isoflurane, and monitoring of oxygen saturation and heart rate. Twelve‐centimeter incision was performed to enter the abdominal cavity, and the fascia of the right abdominal wall peritoneum was excised to create a 6 cm × 4 cm peritoneal defect. The ileocecal region and the contiguous small intestine (80 cm) were placed on dry gauze for 5 min to dry the serous membrane. Both sides of the small intestine were mechanically grazed with a surgical brush for repeated 40 times. Then, ≈600 µL anhydrous alcohol was dropped in the injury area to cause chemical damage. The bleeding bowel segment and the damaged abdominal wall were sutured together with 4‐0 silk. Finally, the abdominal incision was closed.

### Adhesion Scoring

The cecum‐abdominal adhesion of each rat was evaluated following the score standard,^[^ ^22^ ^]^ and the intra‐abdominal adhesions and clinical scores of the porcine model were according to Zuhlke et al.’s method (Table 1).^[^ ^23^ ^]^ The scores were assessed blindly by two researchers.

### Histopathological Analysis

The adhesion tissues were surgically removed, fixed in 4% paraformaldehyde (PFA) overnight at 4 °C, embedded in paraffin, cut into 4 µm thick sections, and stained with hematoxylin and eosin (H&E), Masson trichrome, immunohistochemical antibody (CD68, MPO, TNF‐α and VEGF, Abcam), and immunofluorescent antibody (F4/80, CD86, CD206, CD68, and Nr4a2, Proteintech). The stained slides were observed under light microscopy (Olympus BX 45, Olympus, Japan) and a Nikon Ti‐U microscope equipped with a CSU‐X1 spinning‐disk confocal unit (Yokogawa) and an EM‐CCD camera (iXon+; Andor). The adhesion area, deposition of collagen, and positive cell number were analyzed by Image J software (version 1.8.0_112, National Institutes of Health, America).

### FIR Irradiation on Rat‐Derived Macrophages

In a sterile environment, SD rats were sacrificed, soaked in 75% alcohol for 5 min, lifted upside‐down, and injected with fetal bovine serum (FBS) free Dulbecco's Modified Eagle's Medium (DMEM, Gibco, 10 566 016) under one side of the abdominal cavity. The rats were placed in a supine position; and their abdomen was gently rubbed for 2–3 min. The abdominal cavity was opened after 5–7 min; then 8–9 mL peritoneal fluid was extracted with a sterile syringe followed by centrifugation at 150 g for 5 min. The cells were washed with phosphate buffer saline (PBS), counted, and the activity of the cells was analyzed. The harvested cells were resuspended with 5 mL of DMEM with 10% fetal bovine serum (FBS, Gibco, 26 140 087) and 1% penicillin/streptomycin in dishes at 37 °C with 5% CO_2_. After 24 h, the suspended and loosely attached cells were removed away; and the steadily attached macrophages were stimulated by 100 ng mL^−1^ lipopolysaccharides (LPS, eBioscience, 00‐4976‐93) for 6 h to induce inflammatory responses. A 60‐min FIR irradiation was given on the macrophages that were finally collected for further analysis. For *siRNA* treatment, the primary rat peritoneal macrophages were transfected with si*Nr4a2* or control *siRNA* (synthesized by RiboBio, China) using Lipofectamine 2000 (Invitrogen, 11668‐027) according to the manufacturer's instructions. Sequences of Nr4a2 *siRNA*s were as follows: *siRNA* (1), 5′‐GGACCTCACCAACACTGAA‐3′; *siRNA* (2), 5′‐CCTCCAACTTGCAGAATAT‐3′; *siRNA* (3), 5′‐GCCGAAATCGTTGTCAGTA–3′. After 48 h, cells were treated with LPS and/or FIR for further analysis.

### Quantitative Reverse Transcription PCR (qRT‐PCR)

Total RNA was isolated using RNeasy Mini Kit (Qiagen, Hilden, Germany) and transcribed into cDNA with the M‐MLV (H‐) Reverse Transcriptase System (Vazyme, R021‐01). qRT‐PCR was performed using AceQ qPCR SYBR Green Master Mix (Vazyme, Q111‐02). The specific primers including: TNF‐α, 5′‐ATGGGCTCCCTCTCATCAGT‐3′(forward) and 5′‐ GCTTGGTGGTTTGCTACGAC‐3′(reverse); TGF‐β, 5′‐AGGGCTACCATGCCAACTTC‐3′(forward) and 5′‐CCACGTAGTAGACGATGGGC‐3′(reverse); t‐PA, 5′‐ CTACAGTGCAAGGAGGCCAA‐3′(forward) and 5′‐GTCTCGGTCTGGGTTTCTGC‐3′(reverse); PAI‐1, 5′‐CGTCTTCCTCCACAGCCATT‐3′(forward) and 5′‐ GTTGGATTGTGCCGAACCAC‐3′(reverse). CXCL9, 5′‐TGCCATGAAGTCCGCTGTTC‐3′(forward) and 5′‐R‐CCTTATCACTAGGGTTCCTCGAA‐3′(forward). Transcript levels were calculated relative to the gene β‐Actin, 5′‐ ATATCGCTGCGCTCGTCGT‐3′(forward) and 5′‐ CATACCCACCATCACACCCTGG‐3′(reverse).

### RNA Sequencing and Data Analysis

Rat macrophages were completely lysed by 1 mL RNAiso (TAKARA, NO9109). The total RNA was purified using an RNA simple total RNA kit (TIANGEN, DP419). The quality of purified RNA was examined by an Agilent 2100 bioanalyzer. Next, NEB Next Ultra RNA Library Prep Kit for Illumina (NEB Next, E7530L) was used for library preparation. Finally, high‐quality samples were pooled with product sizes ranging 300–500 bp in equimolar concentrations, then submitted for 150 bp paired‐read sequencing on the Illumina HiSeq X‐ten.

STAR (v2.7.0f) was used to perform the alignment of clean data to rat reference genome mRatBN7.2 and quantify the reads.^[^ ^24^ ^]^ Then, differential gene expression analysis was performed using the R‐package DESeq2.^[^ ^25^ ^]^ The differential expression analysis statistics including log_2_(fold change) and *p* value were extracted from the DESeq object; genes with log_2_(fold change) > 1 and adjusted *p* value < 0.05 were identified as significantly differentially expressed. The volcano plot was generated using R‐package ggplot2, and the KEGG pathway enrichment analysis was performed using R‐package clusterProfiler.^[^ ^26^ ^]^

### Flow Cytometry Analysis

The macrophages in each group were incubated with Rat BD Fc Block Reagent at 4 °C for 5–10 min, followed by the addition of anti‐F4/80 antibody (Abcam, ab16911), anti‐CD11b antibody (Abcam, ab184307), and anti‐CD86 antibody (Abcam, ab238468). After a 30 min incubation at 4 °C, the macrophages were washed with PBS three times. Then, the cells were fixed with 500 µL of cell fixation buffer (Biolegend, 420 801) for 30 min at room temperature in the dark, and centrifugated at 150 g × 5 min. After removal of the supernatant, the cells were thoroughly washed with 500 µL of BD Cytofix/Cytoperm Fixation and Permeabilization wash buffer (BD, 554 722), treated with 100 µL Intracellular staining Permeabilization wash buffer, and incubated with anti‐CD206 antibody (Proteintech, 18704‐1‐AP) for 30 min at room temperature in dark. The cells were finally analyzed by flow cytometry.

### Western Blot Analysis

The rat macrophages were lysed in RIPA lysis buffer (Beyotime, China) containing protease inhibitors. The protein concentration was determined using the Omni‐Easy Instant BCA Protein Assay Kit (Epizyme Biotech, China). The extracted proteins were separated by 12% SDS‐polyacrylamide gel electrophoresis (SDS‐PAGE) and subsequently transferred to nitrocellulose membranes. After blocking with 5% non‐fat dry milk for 2 h, the appropriate primary antibodies against GAPDH, Nr4a1 (Proteintech, 12235‐1‐AP), and Nr4a2 (Proteintech, 10975‐2‐AP) were incubated with membranes overnight. Finally, the signals of proteins were detected by a chemiluminescence system (Bio‐Rad, CA) and analyzed using Image J software.

### Recombinant Adenovirus Construction and Infection in Rats

The adenovirus expressing shRNA for rat Nr4a2 was generated by transfecting the adeno‐associated virus serotype 9 (AAV9) vector pAV‐F4/80‐GFP‐miR30‐sh*Nr4a2* (Vigene Inc., Shandong, China); and sequences for rat Nr4a2‐shRNA was as following: 5′‐CCGCCGAAATCGTTGTCAGTATAGTGAAGCCACAGATGTATACTGACAACGATTTCGGCGGC‐3′. AAV9 vector pAV‐F4/80‐GFP‐miR30‐shRNA contained a nonsense shRNA insert was applied as a negative control. To produce adenoviruses, the constructs were then packaged and transfected into HEK293 cells for 72 h and the viral particles containing supernatants were collected for in vivo experiments.

The rat model of surgical adhesion formation was established and divided into four groups. The control + *shNr4a2* and FIR + sh*Nr4a2* animals were injected intraperitoneally with 500 µL of pAV‐F4/80‐GFP‐miR30‐sh*Nr4a2* AAV9 virus (a titer of 4 × 10^11^ v.g. mL^−1^) per rats every 2 days for the next 7 days after operation. The control and FIR groups were injected intraperitoneally with the negative control AAV9 adenovirus under the same conditions. For the FIR‐treated group, rats were exposed to FIR irradiation for 7 days (10 h per day). All the animals were sacrificed on day 7 post‐operation, and the adhesions were evaluated and collected for further analysis.

### Statistical Analysis

Continuous variables were summarized by means of standard deviations (SD). The comparisons of continuous variables were performed by a two‐tailed Student's *t*‐test or one‐way ANOVA test (also described in figure legend). A *p* value of < 0.05 was considered statistically significant.

## Conflict of Interest

The authors declare no conflict of interest.

## Supporting information

Supporting Information

Supplemental Video 1

Supplemental Video 2

Supplemental Video 3

Supplemental Video 4

## Data Availability

The data that support the findings of this study are available from the corresponding author upon reasonable request.
