# Supplementary material for: A Graphene Composite Film Based Wearable Far‐Infrared Therapy Apparatus (GRAFT) for Effective Prevention of Postoperative Peritoneal Adhesion
Source: Adv Sci (Weinh). 2024 Mar 25;11(22):2309330. doi: 10.1002/advs.202309330 (PMC11165485; doi:10.1002/advs.202309330)
Supplement: Supplementary file 1 — Supporting Information [file ADVS-11-2309330-s004.pdf]

ADVANCED SCIENCE
Open Access

Supporting Information

for *Adv. Sci.*, DOI 10.1002/advs.202309330

A Graphene Composite Film Based Wearable Far-Infrared Therapy Apparatus (GRAFT) for Effective Prevention of Postoperative Peritoneal Adhesion

Xiaohuan Lu, Luming Xu, Yu Song, Xiangnan Yu, Qilin Li, Feng Liu, Xiaoqiong Li, Jiangbo Xi, Shuai Wang*, Lin Wang* and Zheng Wang**

Supporting Information

A Graphene Composite Film Based Wearable Far-infrared Therapy Apparatus (GRAFT) for Effective Prevention of Postoperative Peritoneal Adhesion

Xiaohuan Lu, Luming Xu, Yu Song, Xiangnan Yu, Qilin Li, Feng Liu, Xiaoqiong Li, Jiangbo Xi, Shuai Wang*, Lin Wang*, Zheng Wang**

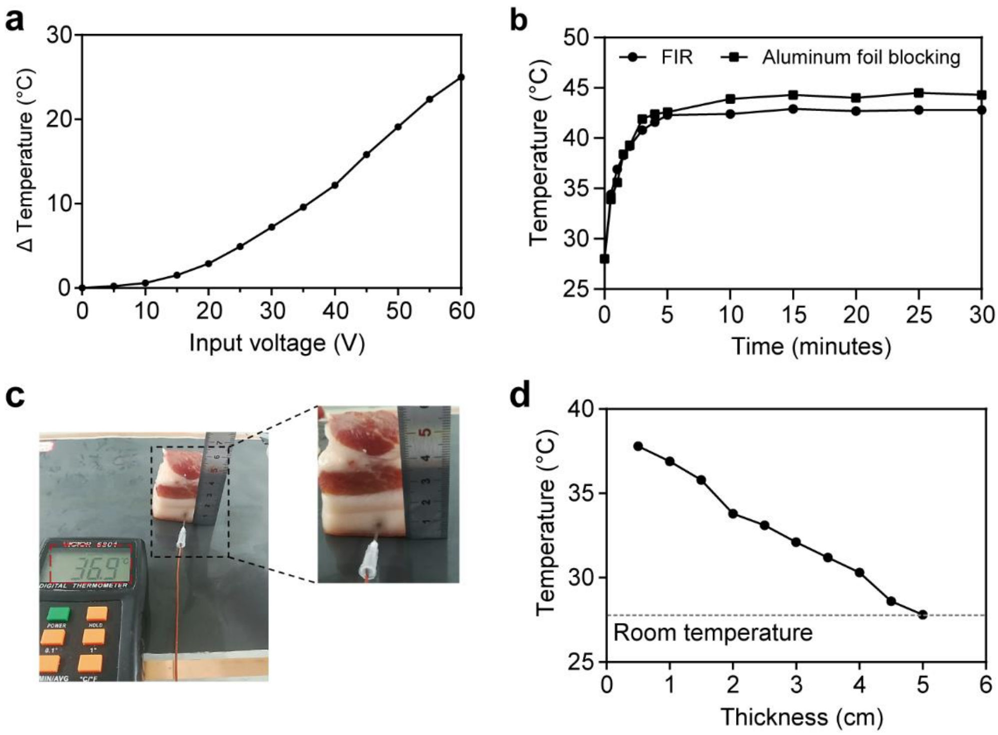

Figure S1. Temperature arising behaviors of the graphene film. a) The temperature-changing profile of a 30 cm × 35 cm F-GCF at different input voltages. b) The heating up curve of a 30 cm × 35 cm F-GCF during a 30-minute power supply (45 V). Aluminum foil blocking: the FIR was blocked using aluminum foil (~10 µm thickness). c) The measurement of the temperature of the pig's abdominal wall. d) The temperatures at different abdominal wall thicknesses with a film temperature of 42 °C (T_{room} = 28 °C).

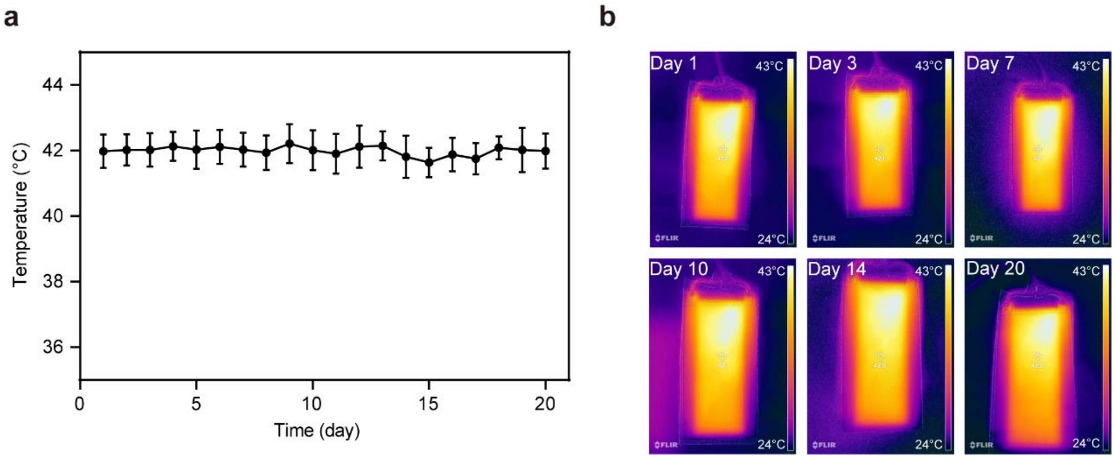

Figure S2. The temperature of F-GCF under DC power supply for a duration of 20 days. a) The temperature of F-GCF on each day. Data are shown as mean ± SD; the temperature was measured 10 times per day. b) The representative thermal imaging graphs of F-GCF on the given day.

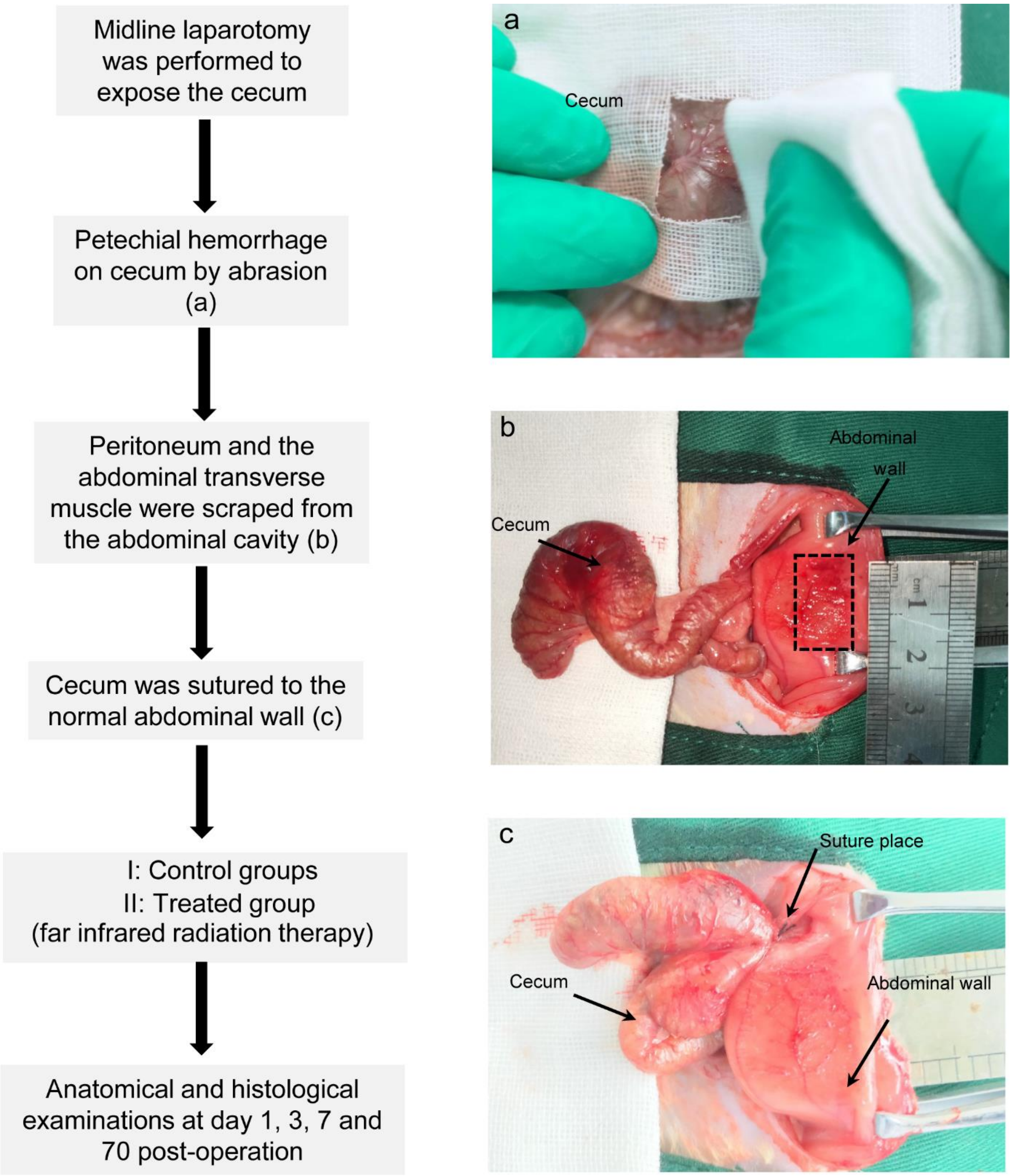

Figure S3. Flowchart and representative photographs of the establishment of rat cecum abrasion-abdominal wall defect model.

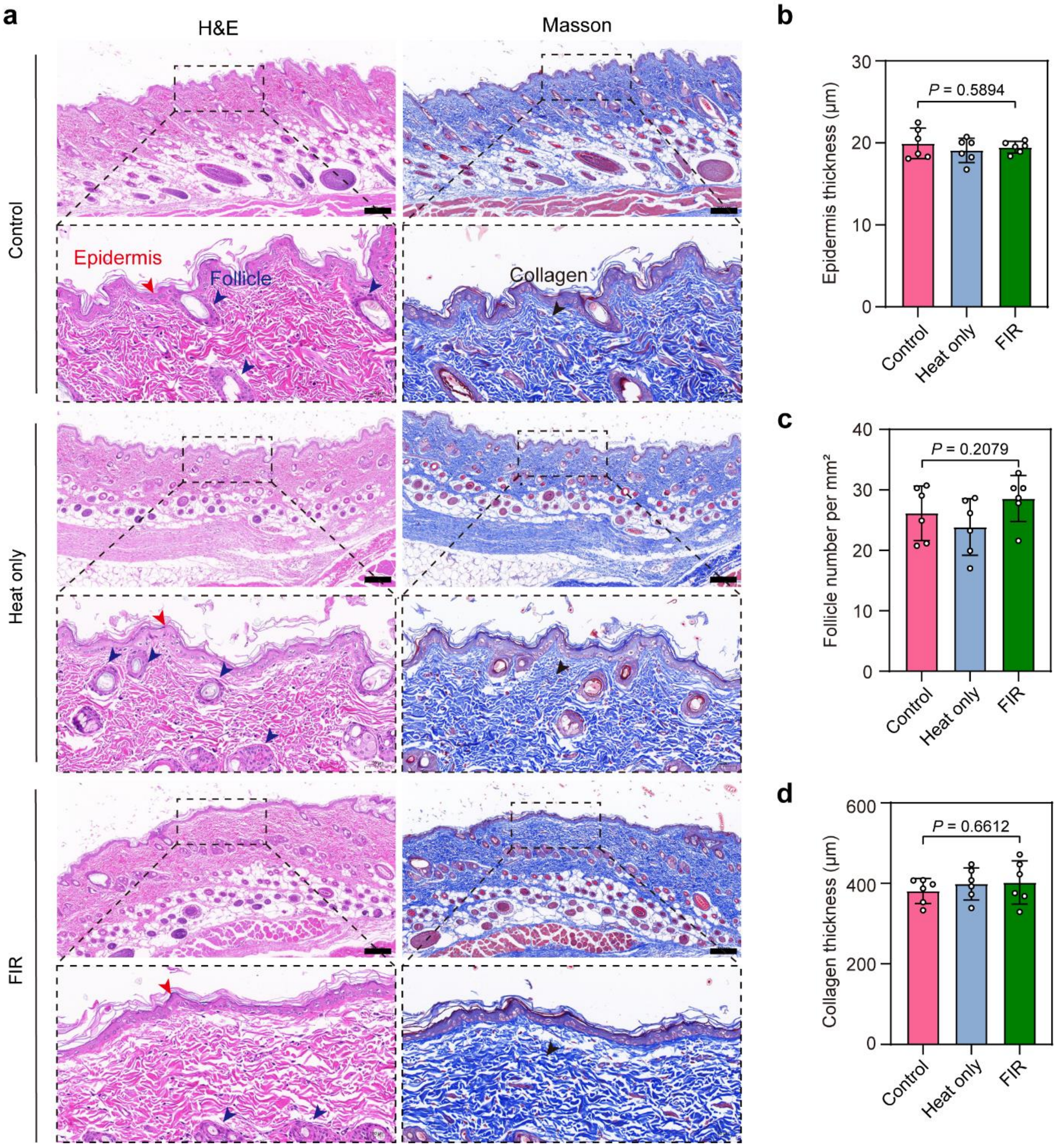

Figure S4. Evaluation of the safety of the FIR generated by F-GCF. a) After being treated by FIR for 7 days, the abdominal skins of rats were sampled and stained by H&E staining. For the "Heat only" group, the FIR was blocked using aluminum foil. Red arrows indicate the epidermis; Blue arrows indicate the follicles; Black arrows indicate collagen. Scar bars, 200 μm. b) Quantitation of the thickness of the epidermis. c) Numbers of follicles in abdomen skin. d) Quantitation of the thickness of the collagen in the abdomen skin. Data are shown as mean ± SD; six rats per group; one image per rat was randomly selected for quantification; *p* values were calculated using one-way ANOVA test.

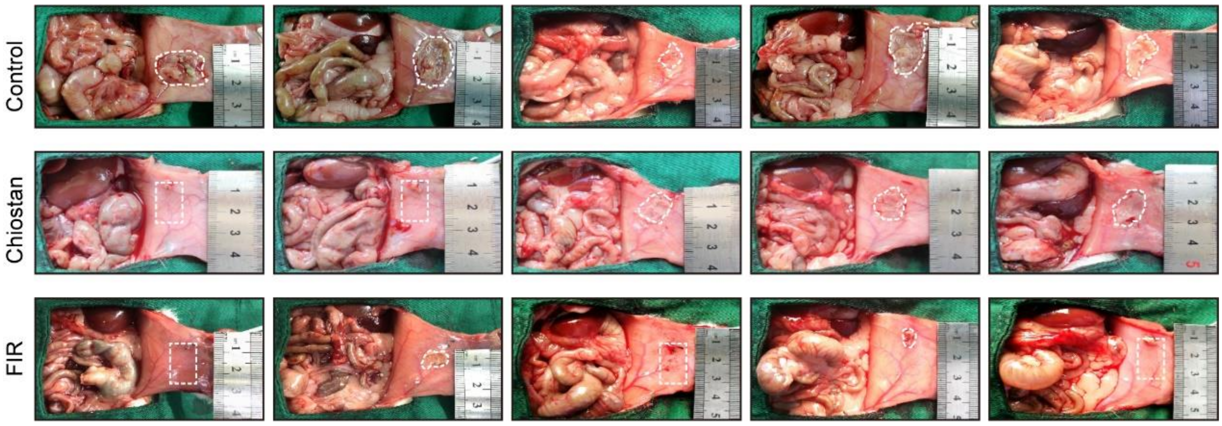

Figure S5. The formation of cecum-abdominal adhesion of each rat on post-operation day 7. The white irregular dotted lines indicate the adhesion area; the white rectangle dotted lines indicate the non-adhesion abdominal wall.

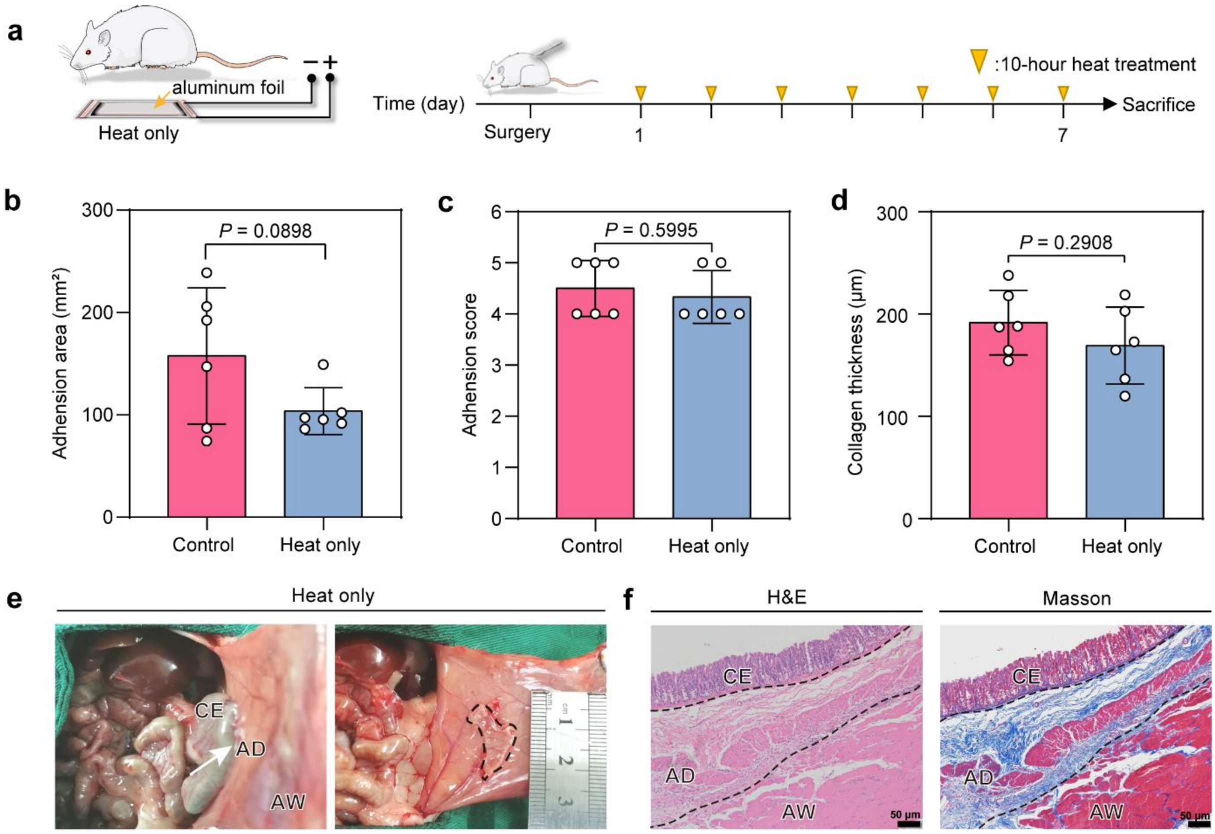

Figure S6. The formation of cecum-abdominal adhesion after heat treatment on post-operation day 7. a) Schematic diagram of the experimental timeline. For the "heat only" (i.e., treated by thermal therapy alone) group, the film temperature was set to 42 °C; and an aluminum foil was covered on the film to block the FIR. b-d) The qualification of b) adhesion area, c) adhesion score, d) collagen deposition thickness on post-operation day 7. e) Representative photographs of the abdominal adhesions in heat-treated rats on post-operation day 7. CE: cecum; AD: adhesion; AW: abdominal wall. f) Representative H&E staining and Masson staining images of the adhesion tissues on post-operation day 7. The black dashed lines indicate the boundaries of adhesions. The images of control group are presented in the main text (Figure 2). Scale bars, 50 μm. Data are shown as mean ± SD; six rats per group; *p* values were calculated using two-tailed Student's *t*-test.

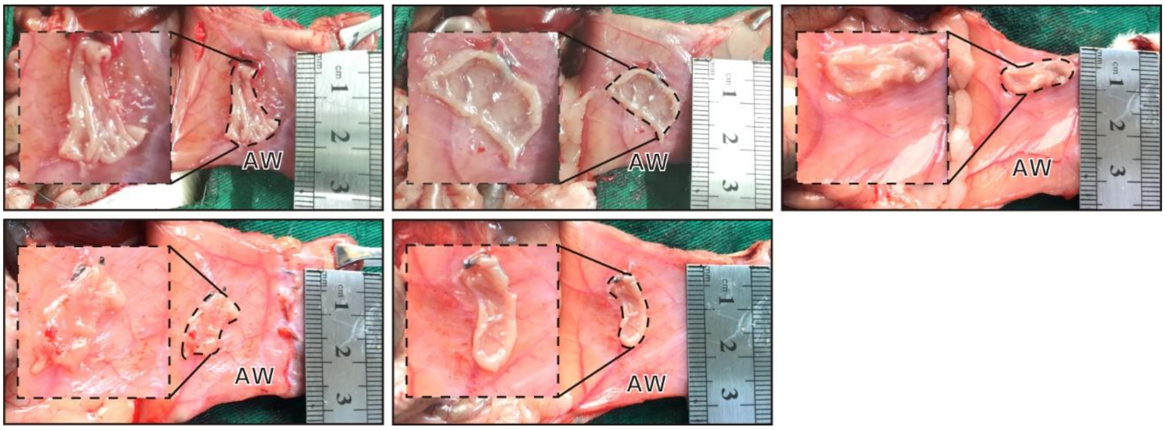

Figure S7. The formation of cecum-abdominal adhesions of the heat-treated rats on post-operation day 7. The images outlined by black dotted lines at the left are the enlargement of the adhesion area. AW: abdominal wall.

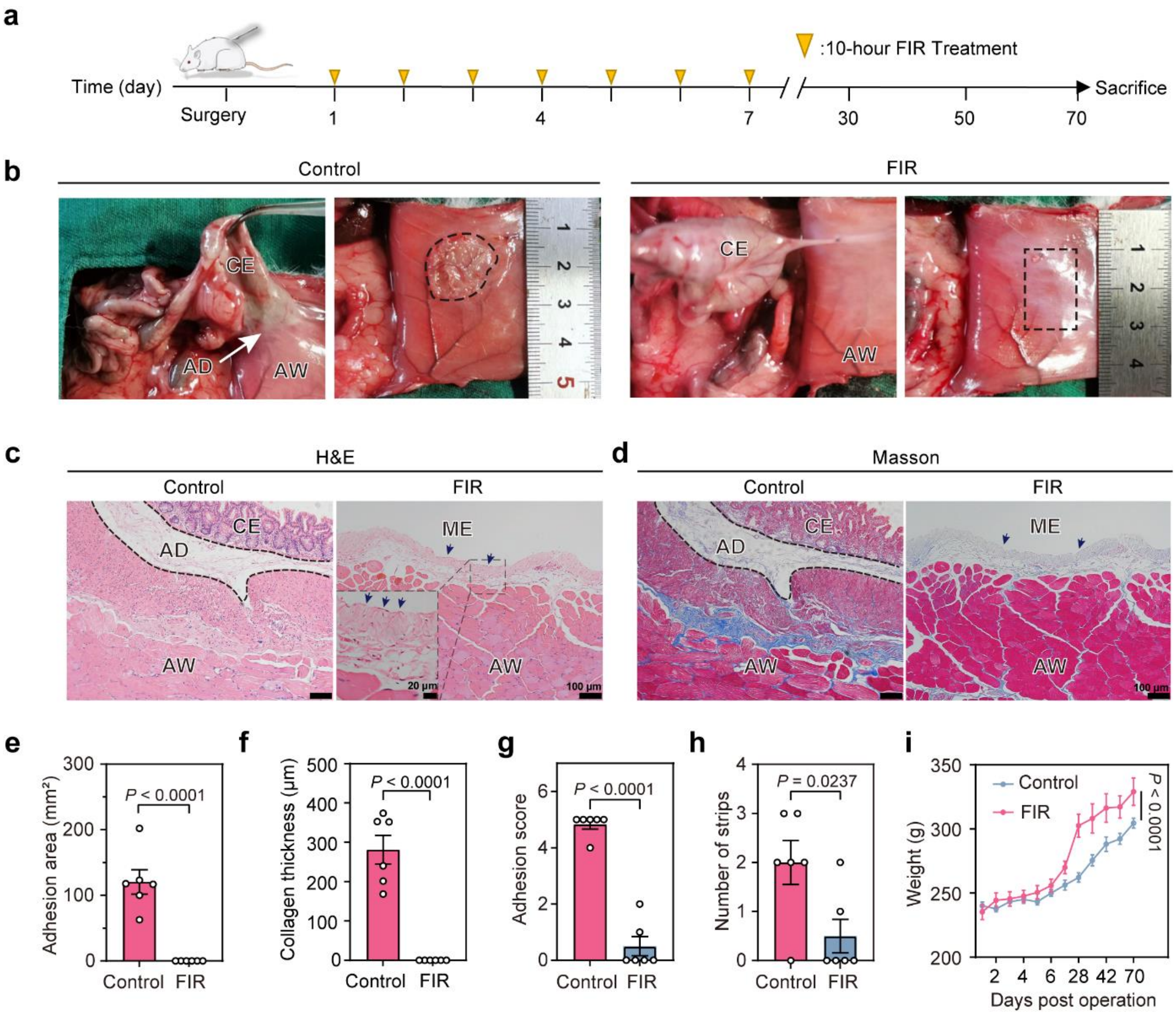

Figure S8. FIR treatment prevents the formation of long-term abdominal adhesion. a) Schematic diagram of the experimental timeline. b) Representative photographs of the abdominal adhesions in FIR-treated or untreated rats on post-operation day 70. CE: cecum; AD: adhesion; AW: abdominal wall. c) Representative H&E staining images of the adhesion tissues on post-operation day 70. The mesothelial monolayer was indicated by the blue arrows and the field is enlarged. The black dashed lines indicate the boundaries of adhesions. Scar bars, 100 μm in the original images, and 20 μm in the enlarged images. d) Representative Masson staining images of the adhesion tissues on post-operation day 70. Scale bars, 100 μm. e-h) The qualification of e) adhesion area, f) collagen deposition thickness, g) adhesion score, and h) the numbers of adhesive strips on post-operation day 70. i) The body weight of the rats. Data are shown as mean ± SD; six rats per group; *p* values were calculated using two-tailed Student's *t*-test.

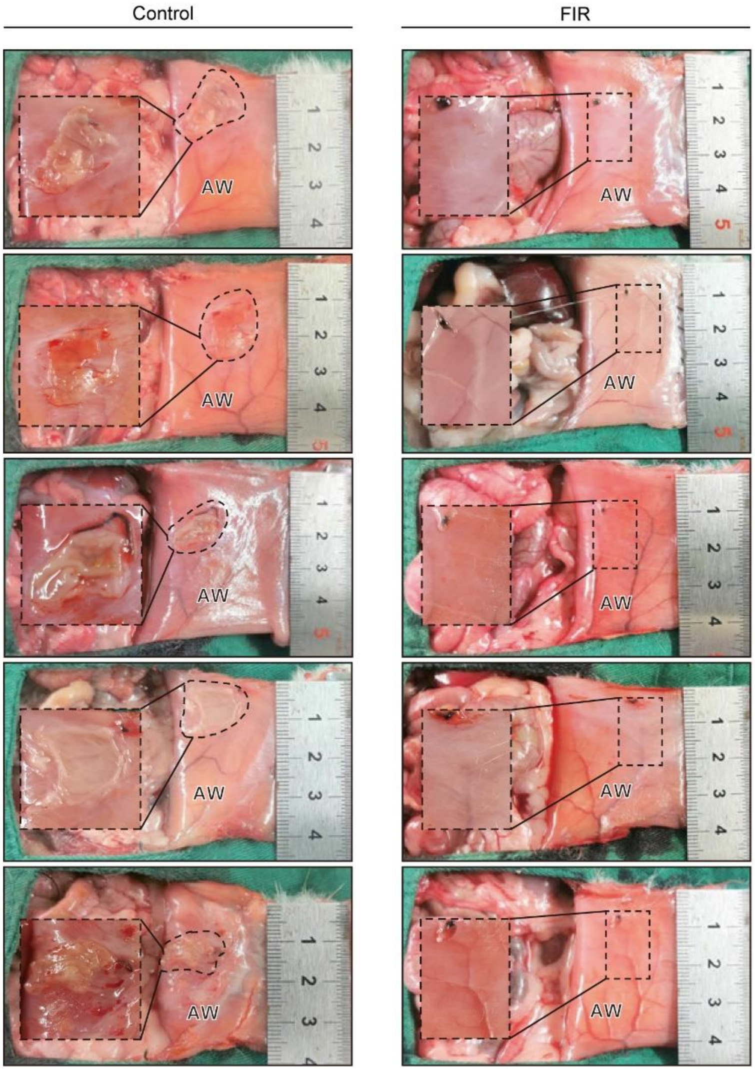

Figure S9. The formation of cecum-abdominal adhesion of each rat on post-operation day 70. The images outlined by black dotted lines at the left are the enlargement of the adhesion area images or non-adhesion abdominal wall images. AW: abdominal wall.

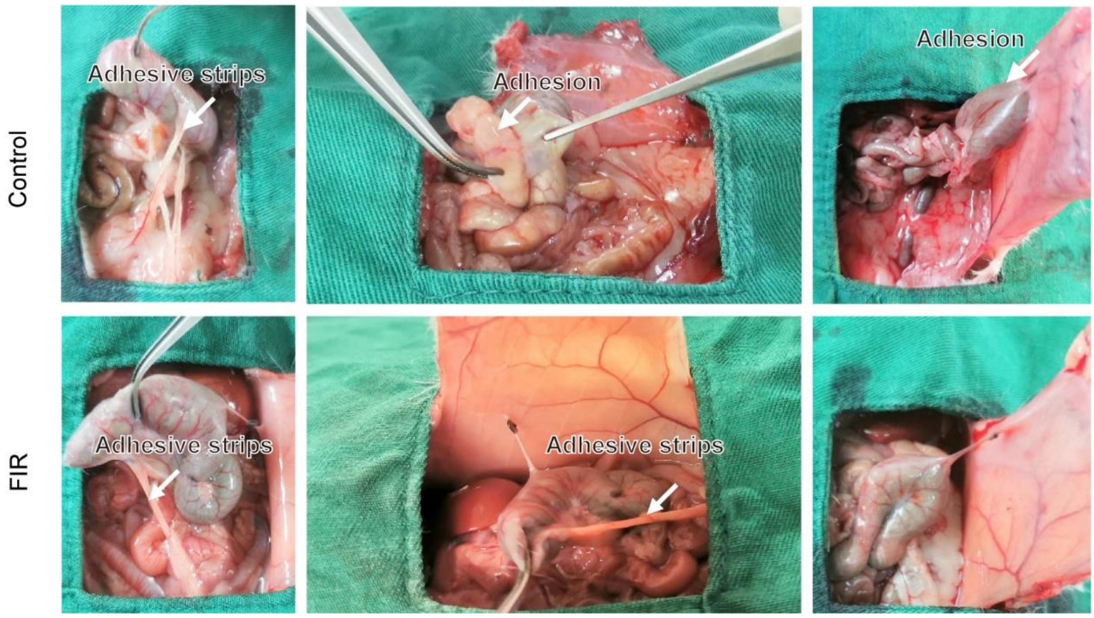

Figure S10. The adhesive strips in the untreated rat and FIR-treated rat at post-operation day 70.

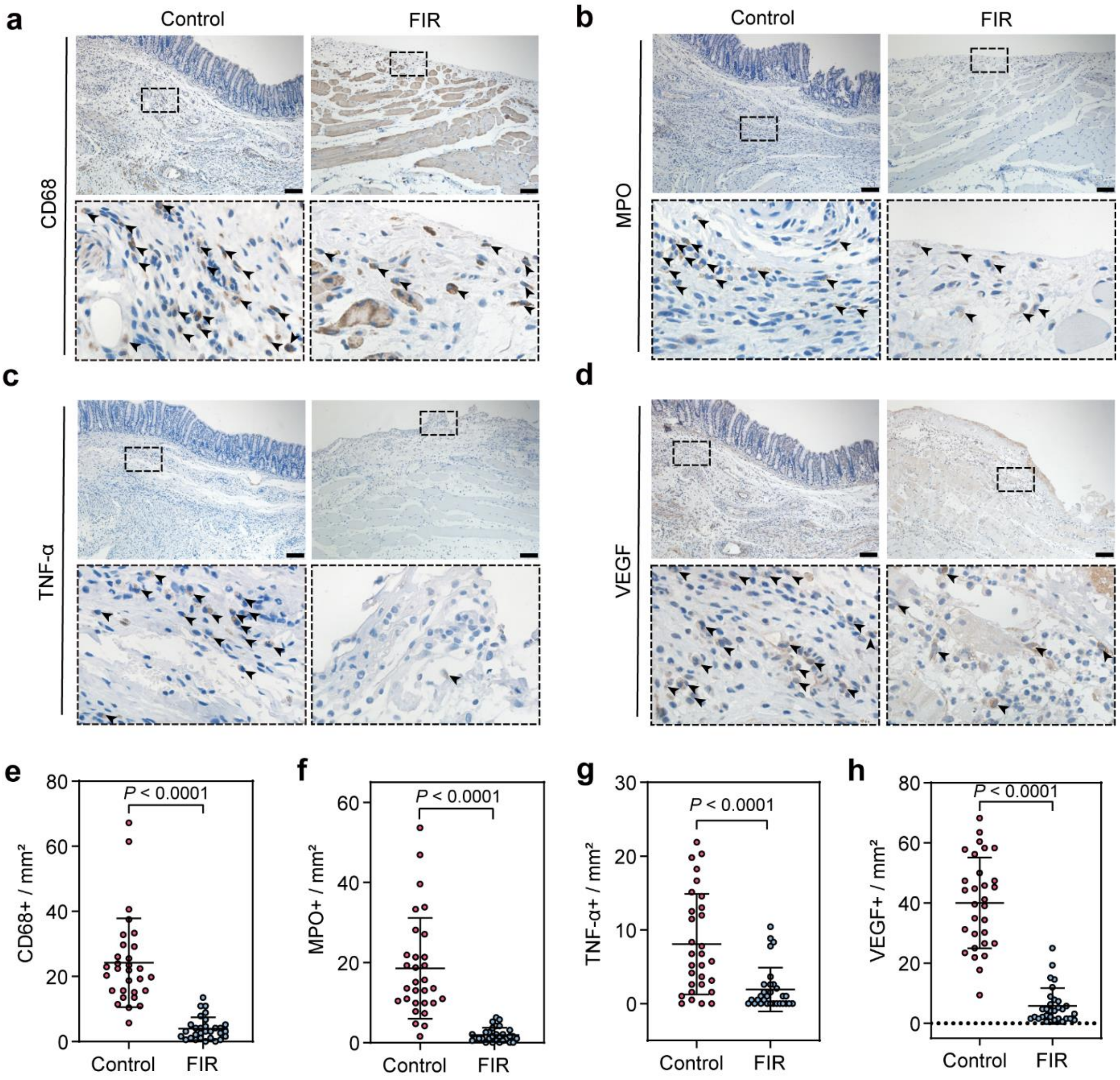

Figure S11. Representative images and quantification of immunohistochemical staining for CD68, MPO, TNF-α, and VEGF in adhesion tissues on post-operation day 7. a-d) Representative immunohistochemical staining images of a) macrophage marker CD68, b) neutrophil marker MPO, c) TNF-α, and d) VEGF in adhesion tissues (or abdominal wall injury site, for rats without adhesions) on post-operation day 7. The black dotted boxes in the upper panel were enlarged in the lower panel. The positive cells in corresponding groups were shown by black arrowheads. Scale bars, 100 µm. e-h) Quantification of e) CD68, f) MPO, g) TNF-α, and h) VEGF positive cells. Data are shown as mean ± SD; six rats per group; five images per rat were randomly selected for quantification; *p* values were calculated using two-tailed Student's *t*-test.

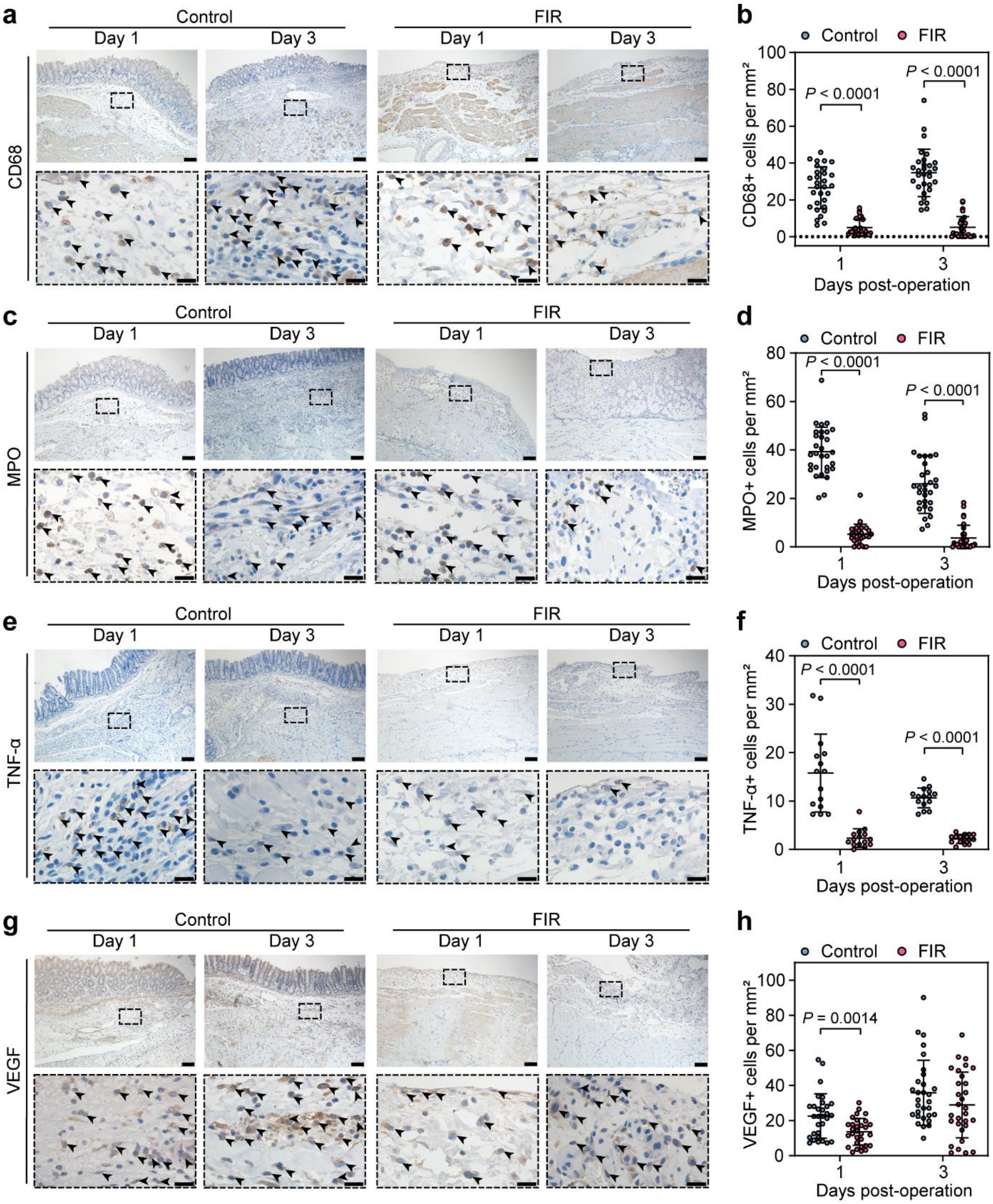

Figure S12. Representative images and quantification of immunohistochemical staining of adhesion tissues on post-operation days 1 and 3. a) Representative images and b) quantification of macrophages (CD68 positive) in adhesion tissues (or abdominal wall injury site, for rats without adhesions) on post-operation days 1 and 3. c) Representative images and d) quantification of neutrophils (MPO positive) in adhesion tissues on post-operation day 1 and 3. e) Representative images and f) quantification of TNF-α in adhesion tissues on post-operation day 1 and 3. g) Representative images and h) quantification of VEGF in adhesion

tissues on post-operation day 1 and 3. The black dashed boxes in the upper panel were enlarged in the lower panel. The positive cells in corresponding groups were indicated by black arrows. Six rats per group; five images per rat were randomly selected for quantification; scale bars, 100 μm for the original images, and 20 μm for the enlarged images; *p* values were calculated using two-tailed Student's *t*-test.

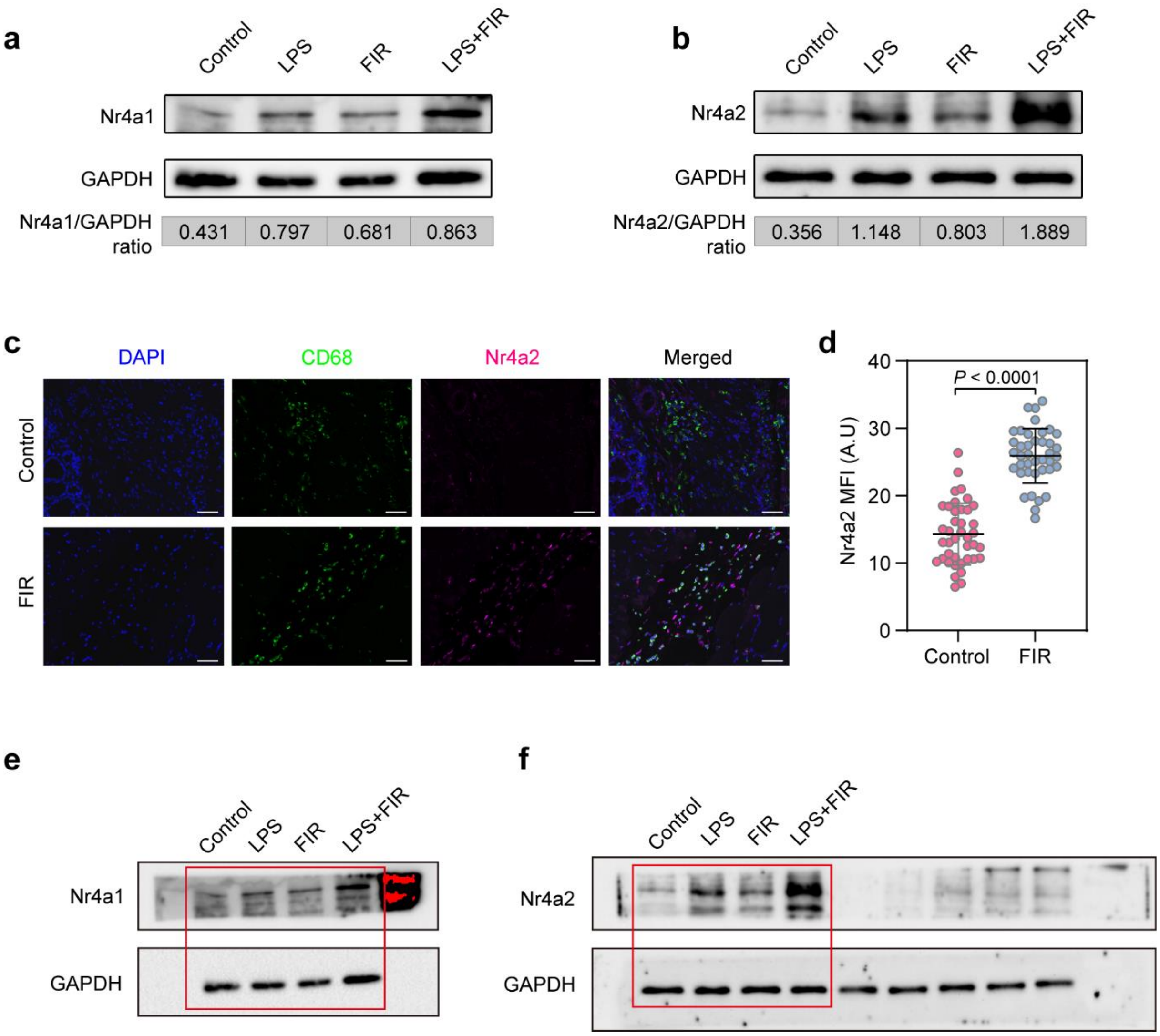

Figure S13. FIR up-regulates the protein expression of Nr4a2. a and b) Western blot analysis of a) Nr4a1 and b) Nr4a2 protein expression in the LPS-treated rat primary peritoneal macrophages with or without FIR irradiation. The relative protein expression level was calculated as the gray intensity ratio of Nr4a1 or Nr4a2 versus GAPDH. c) The representative immunofluorescence staining images of Nr4a2 in the adhesion tissues. Scale bars, 50 μm. d) Quantification of the mean fluorescence intensity (MFI) of Nr4a2. e and f) Raw western blot images of e) Nr4a1 and f) Nr4a2 in the LPS-treated rat primary peritoneal macrophages with or without FIR irradiation. Data are shown as mean ± SD; 40 images from five rats (eight images per rat) in each group were randomly selected for quantification; *p* value was calculated using two-tailed Student's *t*-test.

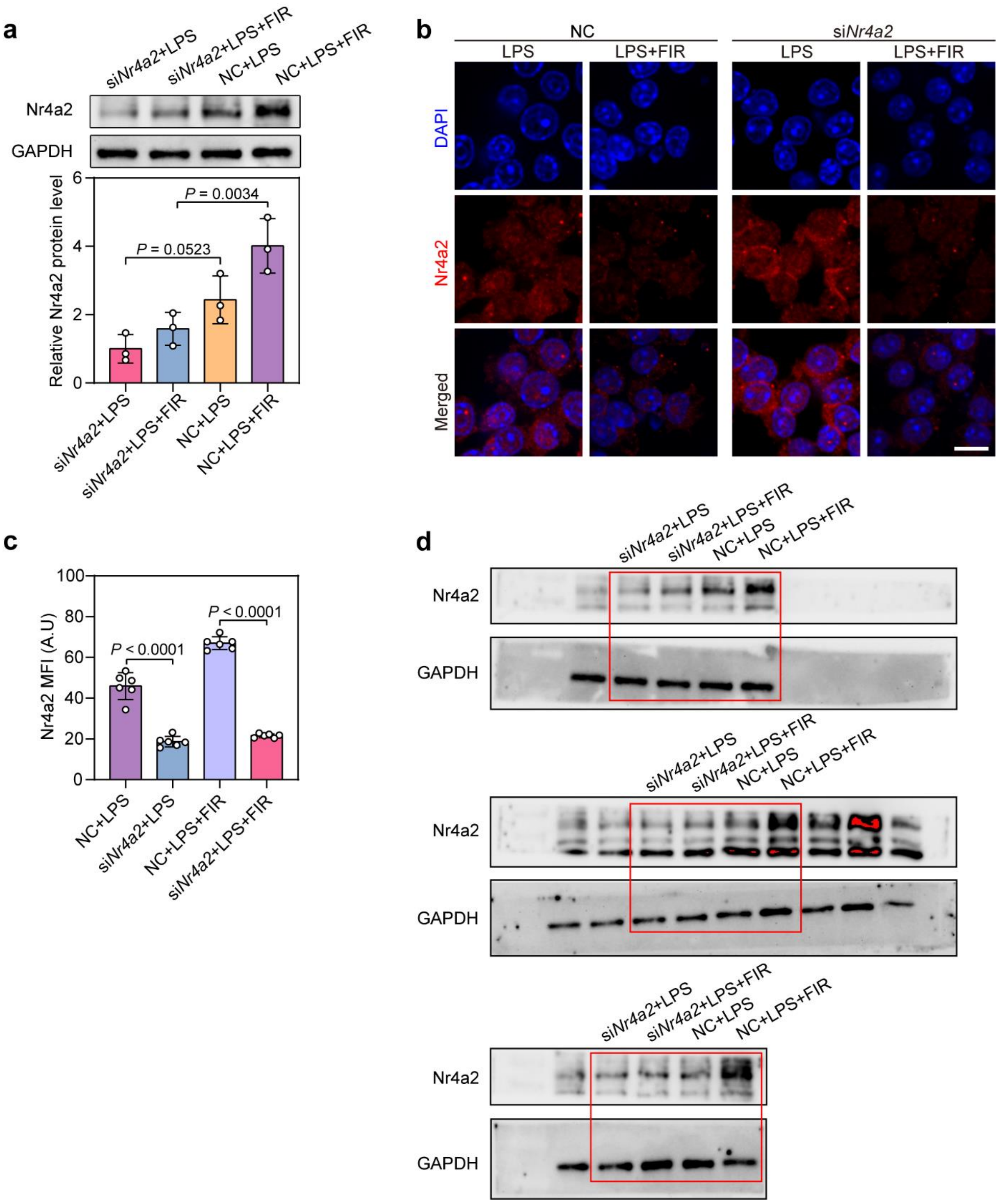

Figure S14. si*Nr4a2* inhibits the expression of Nr4a2 *in vitro*. a) Western blot analysis of Nr4a2 protein expression in the si*Nr4a2* or negative control (NC) siRNA-transfected rat primary peritoneal macrophages. The western blot experiment was repeated three times. b) The representative immunofluorescence staining images of Nr4a2 in the si*Nr4a2* or negative control (NC) siRNA-transfected rat primary peritoneal macrophages. Scale bars, 10 µm. c) Quantification of the mean fluorescence intensity (MFI) of Nr4a2. Six images in each group were randomly selected for quantification. d) Raw western blot images of Nr4a2 protein

expression in the si*Nr4a2* or negative control (NC) siRNA-transfected rat primary peritoneal macrophages. Data are shown as mean ± SD; *p* values were calculated using one-way ANOVA test.

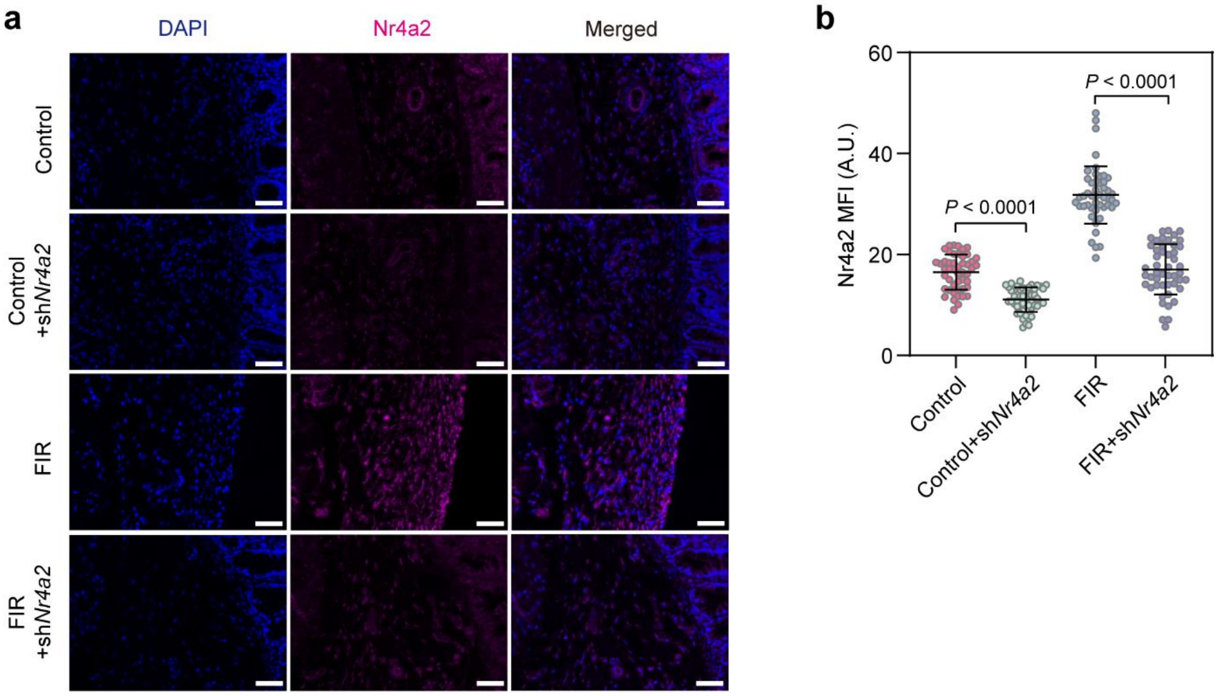

Figure S15. sh*Nr4a2* lentivirus inhibits the expression of Nr4a2 *in vivo*. a) The representative immunofluorescence staining images of Nr4a2 in the adhesion tissues. Scale bars, 50 μm. b) Quantification of the mean fluorescence intensity (MFI) of Nr4a2. Data are shown as mean ± SD; 48 images from six rats (eight images per rat) in each group were randomly selected for quantification; p values were calculated using one-way ANOVA test.

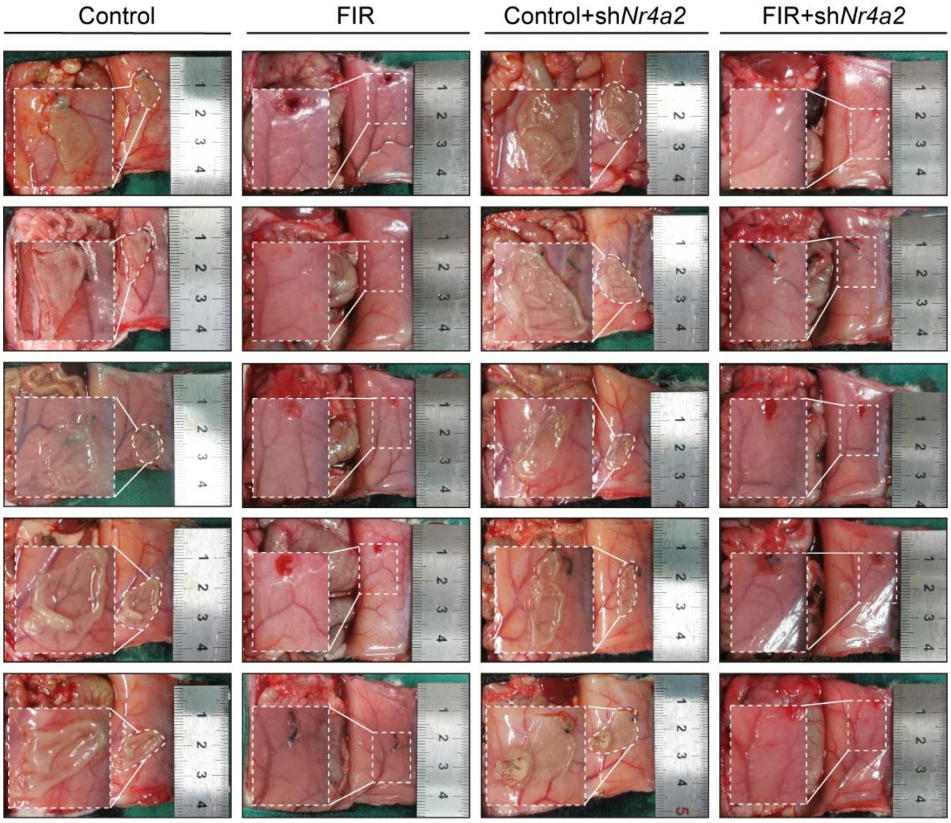

Figure S16. The development of cecum-abdominal adhesion of each rat on day 7 after the indicated treatment. The images enclosed by white dashed lines on the left depict enlarged views of the adhesion area or non-adhesion abdominal wall images.

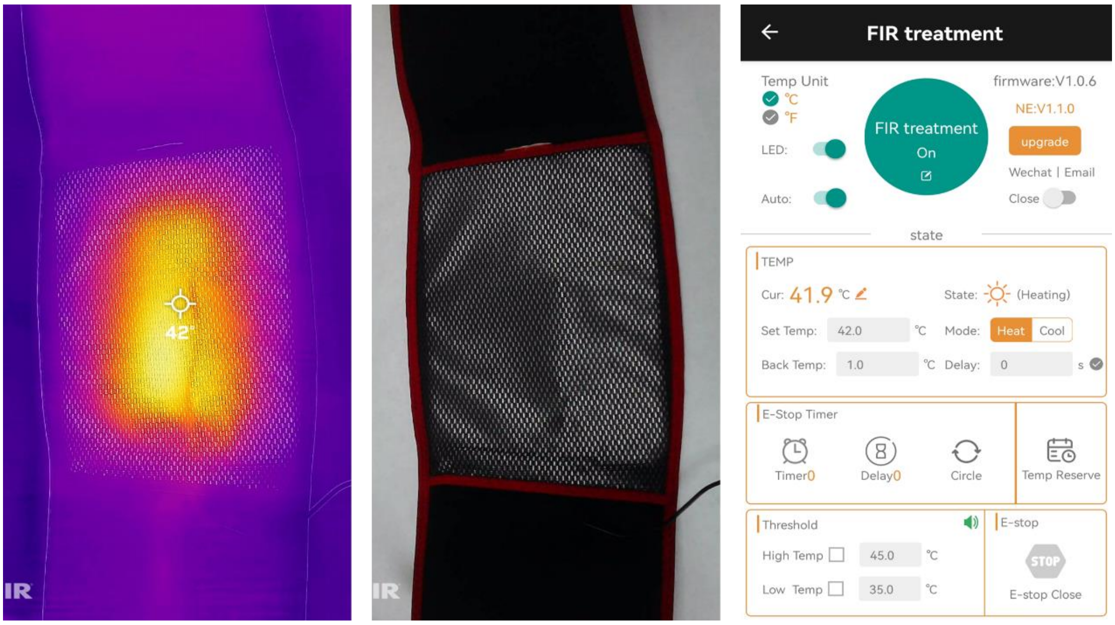

Figure S17. The thermal imaging graph (left), photograph (middle) of a wirelessly controlled, wearable FIR therapy apparatus (GRAFT), and the screenshot of the control software (right) when the treatment temperature was set to 42 °C.

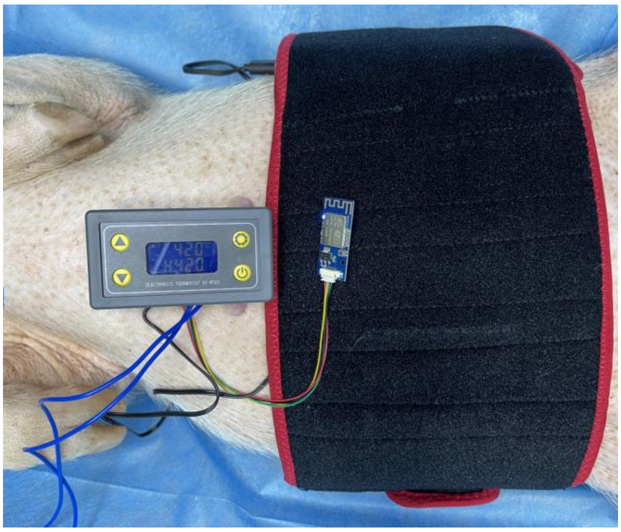

Figure S18. Photograph of a pig with the wearable GRAFT.

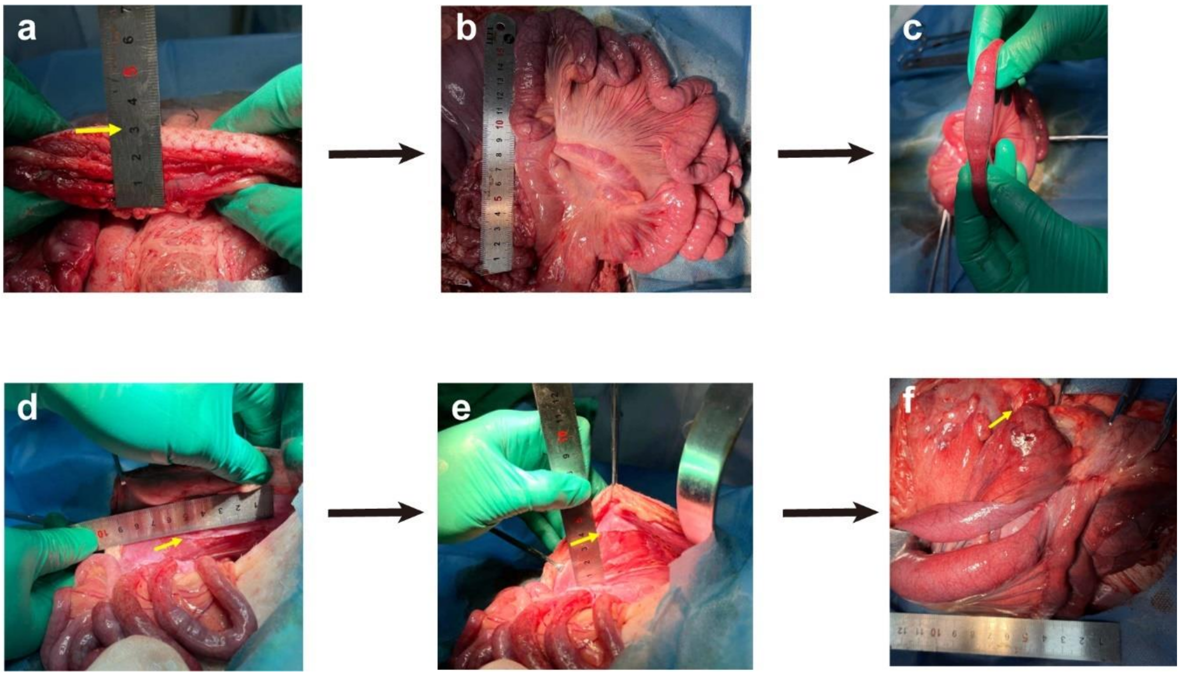

Figure S19. Representative photographs of the establishment of porcine surgical adhesion model. a) A midline abdominal incision was made and the viscera were exposed; b) The contiguous small intestine (80 cm) was placed on dry gauze for 5 minutes; c) The small intestine was mechanically grazed with a surgical brush repeated 40 times until punctate bleeding appeared on the surface of the serosal layer; d,e) The fascia of the right abdominal wall peritoneum was excised to create a 6 cm × 4 cm peritoneal defect; f) The bleeding bowel segment and the damaged abdominal wall were sutured together.

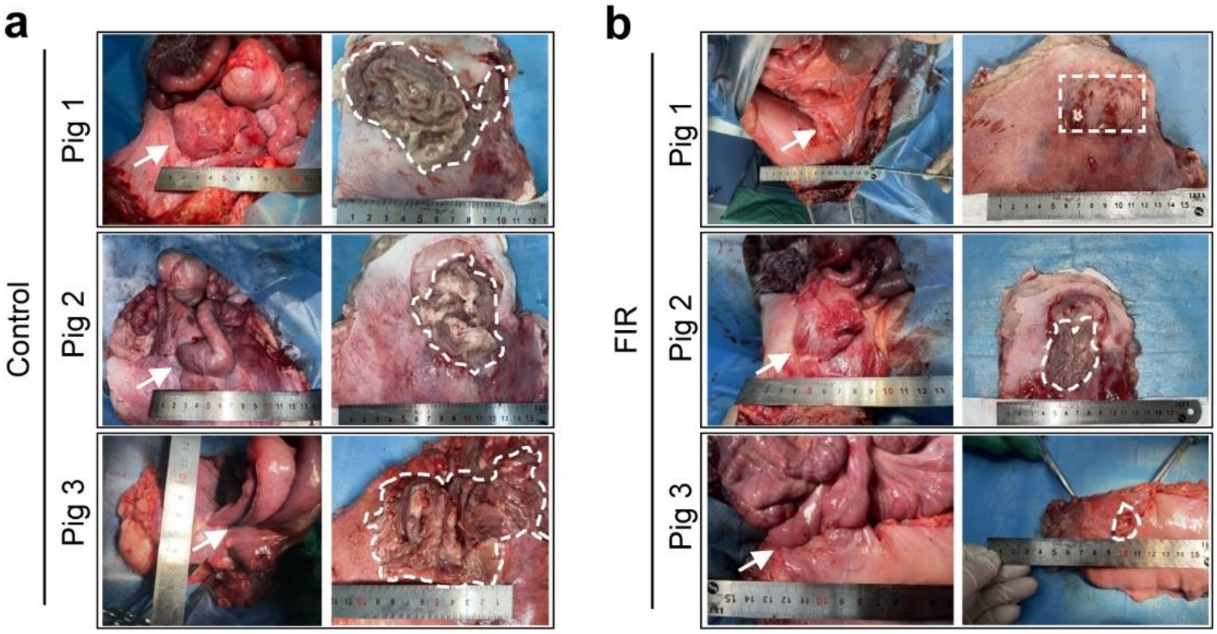

Figure S20. The adhesion in each pig on post-operation day 7. The white irregular dashed lines indicate the adhesion area. The white rectangle dashed lines indicate the non-adhesion abdominal wall.

Video S1.

The flexible property of F-GCF. A 10 cm × 2 cm F-GCF could bend 45° without breaking, suggesting its potential suitability for use in wearable devices.

Video S2.

The work situation of F-GCF based GRAFT. A 20 cm × 15 cm F-GCF based GRAFT (right) was remotely controlled and monitored by smartphone APP software (left). The temperature of F-GCF was increased to approximately 42 °C within six minutes.

Video S3.

Pig wears the F-GCF based GRAFT in the lying state after operation. a 120 cm × 17 cm wearable GRAFT that contained a 20 cm × 15 cm F-GCF was placed around the pig's abdomen, and positioned F-GCF directly against the peritoneal injury area (right). Under the smartphone control and monitor, the temperature of F-GCF was increased to over 40 °C within six minutes (left).

Video S4.

Pig wears the F-GCF based GRAFT in the routine life after operation. The big animal wore the GRAFT that contained a 20 cm × 15 cm F-GCF (right), and treatment temperature of GRAFT was remotely set to 42 °C using an Android smartphone (left), with an FIR emission intensity of 15 mW/cm^2.
